# Effect of antipsychotic drugs on group II metabotropic glutamate receptor expression and epigenetic control in postmortem brains of schizophrenia subjects

**DOI:** 10.1038/s41398-024-02832-z

**Published:** 2024-02-23

**Authors:** Jon DelaCuesta-Barrutia, Oihane Martínez-Peula, Guadalupe Rivero, Jon A. Santas-Martín, Eva Munarriz-Cuezva, Iria Brocos-Mosquera, Patricia Miranda-Azpiazu, Rebeca Diez-Alarcia, Benito Morentin, William G. Honer, Luis F. Callado, Amaia M. Erdozain, Alfredo Ramos-Miguel

**Affiliations:** 1 Department of Pharmacology, University of the Basque Country, UPV/EHU, Leioa, Spain; 2 Biocruces Bizkaia Health Research Institute, Barakaldo, Spain; 3 Centro de Investigación Biomédica en Red de Salud Mental (CIBERSAM), Leioa, Spain; 4 Basque Institute of Legal Medicine, Bilbao, Spain; 5 Department Psychiatry, University of British Columbia, Vancouver, BC Canada

**Keywords:** Schizophrenia, Molecular neuroscience

## Abstract

Antipsychotic-induced low availability of group II metabotropic glutamate receptors (including mGlu_2_R and mGlu_3_R) in brains of schizophrenia patients may explain the limited efficacy of mGlu_2/3_R ligands in clinical trials. Studies evaluating mGlu_2/3_R levels in well-designed, large postmortem brain cohorts are needed to address this issue. Postmortem samples from the dorsolateral prefrontal cortex of 96 schizophrenia subjects and matched controls were collected. Toxicological analyses identified cases who were (AP+) or were not (AP-) receiving antipsychotic treatment near the time of death. Protein and mRNA levels of mGlu_2_R and mGlu_3_R, as well as *GRM2* and *GRM3* promoter-attached histone posttranslational modifications, were quantified. Experimental animal models were used to compare with data obtained in human tissues. Compared to matched controls, schizophrenia cortical samples had lower mGlu_2_R protein amounts, regardless of antipsychotic medication. Downregulation of mGlu_3_R was observed in AP- schizophrenia subjects only. Greater predicted occupancy values of dopamine D_2_ and serotonin 5HT_2A_ receptors correlated with higher density of mGlu_3_R, but not mGlu_2_R. Clozapine treatment and maternal immune activation in rodents mimicked the mGlu_2_R, but not mGlu_3_R regulation observed in schizophrenia brains. mGlu_2_R and mGlu_3_R mRNA levels, and the epigenetic control mechanisms did not parallel the alterations at the protein level, and in some groups correlated inversely. Insufficient cortical availability of mGlu_2_R and mGlu_3_R may be associated with schizophrenia. Antipsychotic treatment may normalize mGlu_3_R, but not mGlu_2_R protein levels. A model in which epigenetic feedback mechanisms controlling mGlu_3_R expression are activated to counterbalance mGluR loss of function is described.

## Introduction

Randomized controlled trials of antipsychotic medications targeting metabotropic glutamate receptors (mGluRs) are plagued by failures of replication [1]. As understanding of the pathophysiology of schizophrenia improves, consideration of clinical features such as genetic background, age of onset, differences between acute and stable phases of illness, and the type and duration of treatment are beginning to be considered as contributors to variability in outcomes of clinical trials [2]. Assessing the integrity of glutamatergic neurotransmission in living humans remains challenging. Tissue levels of glutamate can be measured in living humans with magnetic resonance spectroscopy; these may indirectly provide an estimate of the level of glutamate specifically acting as a neurotransmitter [3]. Directly studying mGluRs in patients with schizophrenia using ligands suitable for positron emission tomography is not feasible at present, and multiple exposures to radioisotope ligands for specific receptors will always be limited by safety concerns. Studies in postmortem brain tissue provide a complementary approach that may help make future clinical trial design informative, with replicable results. Concerning clinical features, patients in large-scale trials are rarely treatment-naïve, and may have antipsychotic medications discontinued for a variable period of time or maintained for augmentation with the novel medication. All of these factors may influence the likelihood of consistency of response to a novel agent across trials.

A retrospective analysis of trials testing the antipsychotic efficacy of pomaglumetad methionil (LY2140023), a nonselective agonist of group II mGluRs (which includes mGlu_2_R and mGlu_3_R), provides an illustration of how confounding variables influence study outcomes. A patient history of antipsychotic drug treatment resulting in high occupancy of serotonin 5-HT_2A_ (5HT_2A_R) as well as dopamine D_2_ (D_2_R) receptors was associated with poor response when compared with patients previously exposed only to predominant D_2_R antagonists [4]. Support for a mechanism related to these observations is provided by preclinical studies comparing the effects of first-(FGA) versus second-(SGA) generation antipsychotic drugs, with higher affinities for D_2_R or 5HT_2A_R, respectively [5, 6]. mGlu_2_R density and mRNA expression was downregulated in rodent brains following chronic clozapine (but not haloperidol) treatment, an effect mediated by histone deacetylase 2 (HDAC2) stimulation, and selective hypoacetylation of histones bound to the *GRM2* gene promoter [7–9]. A contribution of previous antipsychotic drug treatment to downregulation of mGlu_2_R density may impair the capacity to respond to mGlu_2/3_R agonists.

The opportunity to investigate pre-existing treatment effects on multiple receptor types may be a value of postmortem studies. Antipsychotic drugs can be detected in postmortem brain tissue, allowing comparison of receptor amounts in groups of samples with or without antipsychotic drugs present, as well as with samples from individuals with no brain disorders. As well as detection, quantification of antipsychotic drugs allows modeling of the free and protein bound distribution of drugs [10]. With knowledge of the affinity of different antipsychotic drugs for their target receptors, estimates of free drug concentration can in turn provide estimates of receptor occupancy as predictors for effects on novel receptors [11].

Improving consistency across postmortem brain studies of group II mGluRs in schizophrenia (summarized in Supplemental Table S1) also requires attention. Design and technical differences such as selection of brain regions, use of radioligands that detect both mGlu_2_R and mGlu_3_R, or non-validated antibodies to quantify receptor protein levels, and the inconsistent correlation between protein and mRNA levels [12], may all contribute to inconsistencies. Of potentially greatest impact on clinical trial design is the role of previous or concurrent antipsychotic drug treatment, which is sometimes considered as a potential confound, but remains unstudied as a primary predictor of mGluR levels. Few studies addressed mGlu_2/3_R epigenetic regulation, which may mediate the effects of both neurodevelopmental risk factors and antipsychotic medication [13, 14].

The present study investigated the regulation of group II mGluRs in schizophrenia brain samples from a broad perspective. We quantified mGlu_2_R and mGlu_3_R protein immunodensities, mRNA levels, and the load of histone posttranslational modifications (HPTMs) at the *GRM2* and *GRM3* genes in a relatively large case-control postmortem brain cohort of well-characterized schizophrenia cases with qualitative and quantitative assessment of antipsychotic drugs in blood and brain tissue samples, respectively. We specifically evaluated changes in the dorsolateral prefrontal cortex (DLPFC) in the context of well-replicated clinical findings of dysfunction of this brain region in patients with schizophrenia [15–18]. For mGlu_2_R and mGlu_3_R immunodetection, only knockout (KO)-validated antibodies were used. The immunodensities of other G protein-coupled receptors (GPCRs), including the cannabinoid CB_1_ receptor (CB_1_R) and D_2_R, were also estimated to test the internal consistency across our prior studies using a different case-control cohort [19]. Animal studies were used to further explore the impact on brain GPCR densities of both chronic antipsychotic treatment and a neurodevelopmental insult that could lead to increased risk for schizophrenia. Finally, a model of mGlu_2/3_R regulation is proposed to explain the findings of the present and prior studies in the DLPFC of unmedicated and medicated subjects with schizophrenia.

## Materials and methods

### Postmortem human brain samples

Grey matter specimens from the DLPFC (approximating Brodmann area 9), were collected at autopsies in the Basque Institute of Legal Medicine as described elsewhere [20], and immediately stored at −80°C until assayed. Sample collection met all legal and ethical requirements of Spanish legislation; the UPV/EHU Ethical Committee Board for Human Research reviewed all procedures described herein (CEISH-M10/2018/283). For the present study, two case-control cohorts were recruited using identical inclusion/exclusion criteria (see Supplemental Methods). Subjects’ demographic and toxicological features are summarized in Table 1 (collective data) and Supplemental Table S2 (individual characteristics). Samples from Cohort 1 (*N* = 21 case-control pairs) and Cohort 2 (*N* = 27 case-control pairs) were used in all Western blot (WB) assays. Due to limitations in tissue availability, other exploratory experiments used either Cohort 1 or 2. Antemortem schizophrenia diagnoses were performed by psychiatrists according to DSM-IV or ICD-10 criteria, as recorded in medical histories. Each schizophrenia case was paired with a comparison (control) subject without evidence of mental or neurological disorders in their medical records, and similar sex, age, postmortem interval (PMI), and storage time. Routine toxicological assessments in plasma samples detected antipsychotic drugs in 30 schizophrenia cases (AP + ), whereas 18 were antipsychotic-free (AP-) at the time of death. Of note, SGAs (associated with high 5HT_2A_R occupancy) were present in 28 of 30 AP+ subjects (Supplemental Table S2), which did not allow comparisons between FGA and SGA effects. Further quantitative toxicological assessments in cerebellum were performed as previously described [21], using available samples from mainly Cohort 2 (Supplemental Table S2). These measures of total drug concentration were used to model predicted free drug in brain, allowing an estimate of D_2_R and 5HT_2A_R occupancy to be made (see Supplemental Methods).

**Table 1:** Demographic characteristics and blood toxicological data of control and schizophrenia (SZ) subjects by cohort^a^.

	Cohort 1		Cohort 2		Combined	
Diagnosis group	Control	SZ	Control	SZ	Control	SZ
Size, *N*	21	21	27	27	48	48
Females, *N* (%)	7 (33%)	7 (33%)	5 (19%)	5 (19%)	12 (25%)	12 (25%)
Age, years ± SD	43 ± 9	42 ± 9	50 ± 11	50 ± 11	47 ± 10	47 ± 11
PMI, hours ± SD	18 ± 11	16 ± 12	16 ± 7	17 ± 9	17 ± 9	17 ± 10
Brain pH, mean ± SD	6.5 ± 0.2	6.3 ± 0.3	6.5 ± 0.5	6.3 ± 0.4	6.5 ± 0.3	6.3 ± 0.4
Storage time, years ± SD	12 ± 5	12 ± 2	2.6 ± 2.5	4.2 ± 2.8	6.8 ± 6.1	7.5 ± 4.7

Toxicological findings^b^, *N* (%)						
Antipsychotics	None	10 (48%)	None	20 (74%)	None	30 (63%)
Antidepressants	None	None	None	3 (11%)	None	3 (6%)
Mood stabilizers	None	None	None	None	None	None
Benzodiazepines	2 (10%)	11 (52%)	1 (4%)	18 (67%)	3 (6%)	29 (60%)
Ethanol	5 (24%)	None	3 (11%)	None	8 (17%)	None
THC	None	None	4 (15%)	None	4 (8%)	None
Other drugs^c^	2 (10%)	1 (5%)	5 (19%)	none	7 (15%)	1 (2%)

Cause of death, *N* (%)						
Suicide	None	15 (71%)	None	7 (26%)	None	22 (46%)
Natural	7 (33%)	6 (29%)	17 (63%)	15 (56%)	24 (50%)	21 (44%)
Accidental	13 (62%)	None	10 (37%)	3 (11%)	23 (48%)	3 (6%)
Homicide	1 (5%)	None	None	2 (7%)	1 (2%)	2 (4%)

*F* female, *M* male, *PMI* postmortem interval, *SZ* schizophrenia, *SD* standard deviation, *THC* tetrahydrocannabinol.

^a^Individual characteristics are provided in Supplemental Table S2. See Supplement 1.

^b^The presence of psychotropic drugs in brain specimens of Cohort 1 was confirmed by standard toxicological procedures in blood. Brain (cerebellum) toxicological data were also available for all subjects in Cohort 2, and some cases in Cohort 1 (see Supplement 1).

^c^Plasma levels of these drugs were not sufficient to cause intoxication. None of the subjects had history of drug use disorders, according to their medical records.

### Animals and drug treatments

Generation of mGlu_2_R (*Grm2*^−/−^) and CB_1_R (*Cnr1*^−/−^) knockout mice and wildtype littermates was described elsewhere [22, 23]. Chronic treatment of Sprague-Dawley rats (*N* = 10 per group) with haloperidol (1 mg/kg/day), risperidone (1 mg/kg/day) and clozapine (10 mg/kg/day) lasted 21 consecutive days, as reported previously [24]. The maternal immune activation (MIA) model was induced by administration of polyinosinic-polycytidylic acid [poly(I:C), or PIC; 5 mg/kg, i.p.] to pregnant C57BL/6 J mice at gestational day (GD) 9.5, as described before [25, 26]. Dams were maintained undisturbed while breeding, and the offspring (*N* = 7–9 per group) were sacrificed at postnatal day (PND) 84. Pharmacological approaches were done in rats for the complete characterization of the treatment conditions with antipsychotic drugs necessary to obtain clinically comparable occupancy values [27], whereas mice were selected for the MIA modeling to be consistent with our previous work. Cerebral cortices were dissected and immediately frozen to −80°C. All protocols were approved by the UPV/EHU Ethical Committee Board for Animal Research (CEEA - M20/2018/284). Further details on rodent procedures and treatments are provided in the Supplemental Methods.

### Immunoblotting

Preparation of both total homogenates and the pre- and postsynaptic terminal enriched fractions from human and/or rodent cortical samples, electrophoretic separation and transference to nitrocellulose membranes, and quantitative WB procedures were performed as previously described [28, 29], and further detailed in Supplemental Methods. All primary antibodies used are listed in Supplemental Table S3.

### Quantitative PCR

Expression levels of select GPCR mRNA were quantified by reverse transcription PCR (RT-qPCR) analyses as previously described [20]. Standard procedures for mRNA extraction from postmortem human brain tissues from Cohort 1 and conversion into cDNA were used (Supplemental Methods). Forward and reverse primers for mGluRs amplification, as well as the TaqMan probes for GAPDH and RPS13 housekeeping gene expression, are listed in Supplemental Table S4.

### Chromatin immunoprecipitation

Isolation of cell nuclei from postmortem brain samples and chromatin immunoprecipitation (ChIP) assays were done as recently reported [20]. A battery of primary antibodies against trimethylated (Me3) or acetylated (Ac) lysines (K) at histones H3 and H4 (see Supplemental Table S5) was used to immunoprecipitate *GRM2* and *GRM3* gene-attached histones carrying permissive (H3K4Me3, panacetylated H3 [H3panAc], H3K9Ac, H3K27Ac, H4K5Ac, H4K16Ac) or repressive (H3K27Me3) HPTMs. ChIP was followed by amplification of *GRM2* and *GRM3* promoter regions with the primers listed in Supplemental Table S4.

### Data analysis and statistics

WB datasets were first standardized to the mean of the corresponding control group before merging the neurochemical results from both brain cohorts. The distribution of all datasets was tested with the Shapiro-Wilk test. Only those variables estimating the predicted occupancies of D_2_R and 5HT_2A_R displayed non-Gaussian distributions. A multivariate analysis was initially carried out to survey for possible associations between neurochemical measures and potential confounding variables (i.e., age at death, sex, PMI, brain pH, storage time, presence of psychotropic drugs, and/or ethanol). Comparisons between diagnostic groups were addressed following two parallel strategies. According to the case-control design of the study, the primary analysis was a paired *t*-test, either for all subjects together, or after stratifying subject pairs by cohort or by the presence/absence of antipsychotic drugs in blood samples. The secondary approach involved analyses of covariance (ANCOVA), with the neurochemical findings as dependent variables, schizophrenia diagnosis as independent variable, and cohort, sex, age, and PMI as covariates. In this approach, the medication effects were addressed in follow-up analyses replacing the independent variable with a term stratifying subjects in three groups: controls, AP-, and AP+ schizophrenia subjects. Spearman’s correlation analyses initially tested the possible associations between D_2_R or 5HT_2A_R predicted occupancies, and the GPCR immunodensities. Occupancy estimates were further used as independent variables in follow-up, sex-, age-, and PMI-controlled regression models predicting mGluR immunodensities. In animal studies, comparisons between groups were performed using Student’s *t*-test or one-way analysis of variance (ANOVA), followed by Dunnet’s test. All tests were two-tailed, and the statistical significance was set to *p* < 0.05. False discovery rate (FDR; Benjamini–Hochberg method) adjustment was applied whenever appropriate. Data were analyzed with JMP17 (SAS Institute, Cary, NC, USA), and plotted with Prism 9 (GraphPad, La Jolla, CA, USA).

## Results

### Immunodetection of mGlu_2_, mGlu_3_, CB_1_, and D_2_ receptors in human DLPFC and effects of potentially confounding variables

**Fig. 1:**
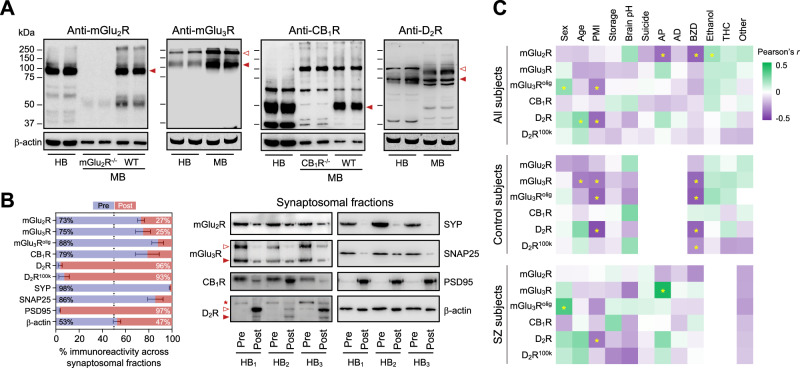
Antibody validation and evaluation of potentially confounding variables. **A** Characterization of the four antibodies selected for quantification of mGlu_2_ (ab15672), mGlu_3_ (ab166608), CB_1_ (ab23703), and D_2_ (AB5084P) receptors (see Supplemental Table S3) in human and rodent brains, and validation in *Grm2* (mGlu_2_R^−/−^) and *Cnr1* (CB_1_R^−/−^) knockout mice. Images show representative immunoblots of human (HB), and wildtype (WT) or knockout mouse (MB) cortical samples loaded in duplicate onto SDS gels, and resolved by standard SDS-PAGE, followed by immunoprobing with the above commercial antibodies. Closed arrowheads indicate those immunoreactive bands considered selective; i.e., bands approaching the theoretical molecular size of the protomeric form of each GPCR, and absent in target knockout mice [knockout validation of anti-mGlu_3_R and -D_2_R antibodies was published elsewhere [30, 31]]. Open arrowheads indicate other bands putatively corresponding to oligomeric (mGlu_3_R^olig^) or glycosylated (D_2_R^100k^) species of the receptors, and were also considered for quantitative assays based on previous validation studies [30, 31]. All membranes were stripped and reprobed with anti-β-actin antibody as a loading control. Molecular masses (in kDa) of SDS-PAGE prestained standards are shown on the left. **B** Horizontal bar plots (left) and immunoblots (right) depicting the distribution of the indicated protein species across the pre-(Pre; blue) and post-(Post; red) synaptic synaptosomal fractions isolated from postmortem human DLPFC of three different control subjects (HB_1/2/3_). The selective (or preferential) detection of synaptophysin (SYP) and synaptosomal-associated protein of 25 kDa (SNAP25) at the presynaptic fraction, and postsynaptic density 95 (PSD95) at the postsynaptic fraction, account for the purity of the subcellular compartments. Closed and open red arrowheads indicate the same as in **A**. The red asterisk in D_2_R immunoblot points at a putative presynaptic D_2_R species of ~150 kDa not consistently observed in crude homogenates, and was not further evaluated. **C** Heatmap representing Pearson’s *r*-coefficients of the pairwise associations between the potentially confounding variables of the study (displayed along the *x*-axis; AD antidepressants, AP antipsychotics, BZD benzodiazepines, PMI postmortem interval, THC tetrahydrocannabinol) and the studied GPCR immunodensities (displayed along the *y*-axis) in postmortem samples of the DLPFC from subjects with schizophrenia (SZ) and controls, combined altogether (top panel) or segregated by diagnosis (middle and bottom panels). Color scale on the *r*-values is shown on the top-right corner. *p < 0.05.

All antibodies used in quantitative immunoblotting experiments were previously validated in brain samples from knockout animals lacking the target receptor (Fig. 1A and Supplemental Fig. S1A) (see also refs. [30, 31]). While the present study mainly focused in the monomeric ~95-kDa mGlu_2_R, ~110-kDa mGlu_3_R, ~50-kDa CB_1_R, and ~75-kDa D_2_R species, other receptor forms (further referred to as mGlu_3_R^olig^ and D_2_R^100k^; Fig. 1A) were also quantified. Synaptosome fractionation experiments showed the preferential location of mGlu_2_R, mGlu_3_R, and CB_1_R at the presynaptic terminals, while ~75 and ~100 kDa D_2_R species displayed selective postsynaptic localization (Fig. 1B). Further considerations concerning antibody selectivity and pre- versus postsynaptic enrichment receptor distribution are reported in the Supplemental Results.

The effect of potentially confounding variables on GPCR immunodensities is reviewed in detail in the Supplemental Results. The paired design of the study accounted for possible age, sex, PMI and storage time effects. Multivariate analyses detected possible effects of benzodiazepine medication on brain GPCR amounts in control subjects (Fig. 1C). However, complementary ANCOVAs adjusting for benzodiazepine presence discarded potential effects of benzodiazepine medication on the reported results (see Supplemental Results).

### Immunodensities of mGlu_2_, mGlu_3_, CB_1_, and D_2_ receptors in schizophrenia DLPFC

**Fig. 2:**
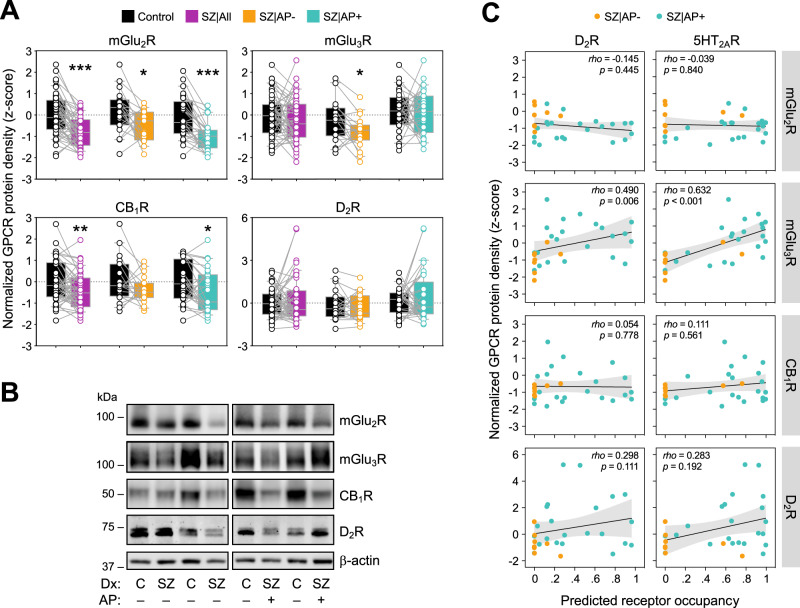
Immunodensiities of target GPCRs in schizophrenia brain samples. **A** Box plots representing β-actin-normalized mGlu_2_R, mGlu_3_R, CB_1_R, and D_2_R immunodensities in the DLPFC of age-, sex- and PMI-matched pairs of schizophrenia (SZ) cases and controls, either altogether (All) or stratified by the absence (AP-) or presence (AP + ) of antipsychotic drugs in the blood sample of the SZ subject pair at the time of death. Paired *t*-tests detected significant differences (*p < 0.05, **p < 0.01, ***p < 0.001) between the diagnostic groups for mGlu_2_R (All, *t*_1,47_ = 5.66; AP-, *t*_1,17_ = 2.63; AP + , *t*_1,29_ = 5.41), mGlu_3_R (AP-, *t*_1,17_ = 2.27), and CB_1_R (All, *t*_1,47_ = 2.98; AP + , *t*_1,29_ = 2.70). **B** Representative mGlu_2_R, mGlu_3_R, CB_1_R, and D_2_R immunoblots depicting samples from all diagnosis (Dx) and toxicological (AP− and AP + ) comparison groups. Molecular mass (in kDa) of the most proximal prestained protein marker (Bio-Rad) to the target proteins is indicated on the left. **C** Scatterplots depicting pairwise associations between the predicted occupancy values of D_2_R and 5HT_2A_R, as estimated from drug concentrations in cerebellum samples, and the immunodensities of mGlu_2_R, mGlu_3_R, CB_1_R, and D_2_R in the DLPFC of SZ subjects. Fit line, as well as *rho-* and *p*-values of the Spearman correlation test are shown for each analysis.

Cortical mGlu_2_R amounts were significantly lower in schizophrenia samples (−31%, *p* < 0.001), as compared to paired sex-, age-, and PMI-matched control samples (Fig. 2A, B). Lower mGlu_2_R immunoreactivity was observed in both AP- (−29%, *p* < 0.05) and AP+ (−33%, *p* < 0.01) schizophrenia subgroups. Direct comparison between AP- and AP+ cases did not yield statistically significant differences (Supplemental Fig. S2A and Table S6). These data suggest that schizophrenia is associated with lower cortical expression of mGlu_2_R protein, and antipsychotic medication has no detectable effect on mGlu_2_R levels.

While schizophrenia and matched control samples did not differ in amounts of monomeric mGlu_3_R, AP- (but not AP + ) schizophrenia cases displayed significantly lower mGlu_3_R immunodensities (−21%, *p* < 0.05), compared to their corresponding control pairs (Fig. 2A). Complementary ANCOVA tests demonstrated a difference between AP- and AP+ subgroups (−34%, *p* < 0.01) (Supplemental Fig. S2A and Table S6). Cortical immunodensities of mGlu_3_R^olig^ species did not differ across the diagnostic groups and subgroups (Supplemental Fig. S2A). These observations indicate that schizophrenia is associated with lower expression of mGlu_3_R monomers in the DLPFC, and antipsychotic medication may ameliorate this deficit.

We also found downregulation of CB_1_R (−17%, *p* < 0.01) in the DLPFC samples of schizophrenia subjects, as compared to matched controls (Fig. 2A, B). While this difference was mainly attributed to AP+ schizophrenia subjects (−19%, *p* < 0.05), subgroup ANCOVA analyses did not detect significant differences between AP- and AP+ cases (Supplemental Fig. S2A and Table S6). Finally, cortical immunodensities of D_2_R species were similar in all groups.

When analyzed separately, Cohorts 1 and 2 displayed a very similar pattern of results to those reported above in terms of GPCR immunodensities across the diagnostic groups and subgroups (Supplemental Fig. S2B), although the statistical significance for some comparisons in these smaller groups was lost. Finally, among AP+ cases, cortical immunodensities of mGlu_2/3_R, CB_1_R, and D_2_R were similar in subjects who committed suicide, compared to those who died from other causes (data not shown).

### Associations between D_2_R and 5HT_2A_R occupancy estimates and cortical immunodensities of target GPCRs in subjects with schizophrenia

Toxicological findings were highly consistent, as all drugs detected in blood were also found in brain samples. Conversely, some drugs detected in brain were not found in blood, likely due to their high liposolubility. For example, two cases initially classified as AP- according to blood toxicology, displayed detectable brain levels of paliperidone (see Fig. 2C), a particularly lipophilic compound. Control subjects were confirmed as being free from antipsychotic drugs, and were therefore excluded from the analyses to avoid the potential confounds of illness-associated variations in the GPCR targets.

To unmask the potential association between D_2_R and/or 5HT_2A_R occupancy and drug-induced alterations of target GPCR amounts in the DLPFC of schizophrenia cases, we estimated D_2_R and 5HT_2A_R occupancies from brain tissue concentrations of drugs with significant affinities for these receptors. Initial Spearman’s correlation analyses revealed that higher occupancy values of both D_2_R and 5HT_2A_R were associated with greater amounts of mGlu_3_R protein (but not other GPCRs) in schizophrenia DLPFC samples (Fig. 2C). More detailed sex-, age-, and PMI-controlled models testing the association between receptor occupancy and mGluR protein levels showed an effect of 5HT_2A_R (but not D_2_R) occupancy on mGlu_3_R (but not mGlu_2_R) immunodensities (Supplemental Table S7). Although the improvement in variance explained in the model when adding both 5HT_2A_R and D_2_R occupancy estimates suggests a minor contribution from D_2_R occupancy as well. These data suggest that antipsychotic-induced blockade primarily of 5HT_2A_R contributes to the upregulation of monomeric mGlu_3_R density in the DLPFC of subjects with schizophrenia.

### Chronic effect of antipsychotic drugs on target GPCR immunodensities in rat cortex

**Fig. 3:**
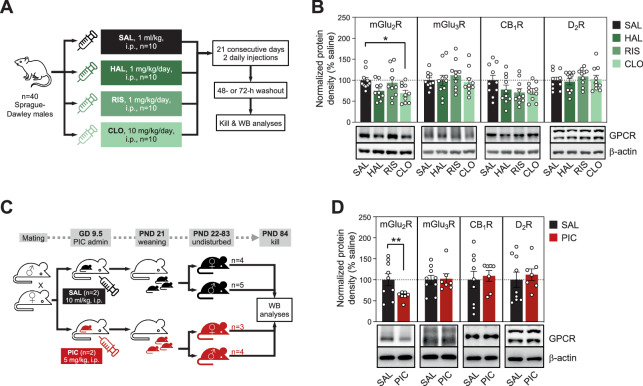
Immunodensities of target GPCRs in rodent brains following antipsychotic treatments and prenatal poly(I:C) exposure. **A** Cartoon illustrating chronic treatment procedures with saline (SAL), haloperidol (HAL), risperidone (RIS), and clozapine (CLO) in rats and further Western blot (WB) analyses. **B** Effects of antipsychotic chronic treatments on cortical immunodensities of mGlu_2_R, mGlu_3_R, CB_1_R and D_2_R. Bars represent mean ± SEM values of each treatment group. One-way ANOVA detected significant differences between treatment groups for mGlu_2_R densities (*F*_3,36_ = 3.29). *p < 0.05, ANOVA followed by Dunnett’s *post hoc* test. **C** Cartoon illustrating the polyinosinic–polycytidylic acid- (PIC) induced maternal immune activation (MIA) procedure in pregnant dams, as compared to saline (SAL) administration, and further WB analyses in the adult offspring. **D** Effects of prenatal exposure to PIC-induced MIA on cortical immunodensities of mGlu_2_R, mGlu_3_R, CB_1_R and D_2_R in adult mice. Bars represent mean ± SEM values of SAL- or PIC-exposed groups of mice. Student *t*-test detected significant differences between treatment groups for mGlu_2_R densities (*t*_1,14_ = 2.27; *p < 0.05).

The potential effects of chronic exposure to antipsychotic drugs on cortical amounts of target GPCRs was further addressed in rats chronically treated with haloperidol, risperidone, or clozapine (Fig. 3A). Notably, clozapine treatment (−31%, *p* < 0.05), and possibly haloperidol as well (−25%, *p* > 0.05), downregulated cortical amounts of mGlu_2_R, but not mGlu_3_R, as compared to saline-treated animals (Fig. 3B). Nonsignificant downregulations of CB_1_R (22–29%, *p* > 0.05) were also observed in the cerebral cortex of rats exposed to all three antipsychotic drugs, while D_2_R immunodensities remained unchanged in the same brain samples (Fig. 3B).

### Effect of prenatal exposure to poly(I:C) on cortical immunodensities of target GPCRs

We used a poly(I:C)-induced murine model of maternal immune activation (Fig. 3C) to test the possibility that the observed glutamatergic alterations in schizophrenia brains might be associated with complications during neurodevelopment, which are well-known risk factors for schizophrenia [32]. Cortical samples of mice prenatally exposed to poly(I:C) displayed lower amounts of mGlu_2_R (−38%, *p* < 0.05), compared to those in saline-exposed animals (Fig. 3D). In contrast, the immunodensities of all other GPCRs studied remained unaltered.

### Gene expression and epigenetic control of mGluRs in the DLPFC of schizophrenia subjects

**Fig. 4:**
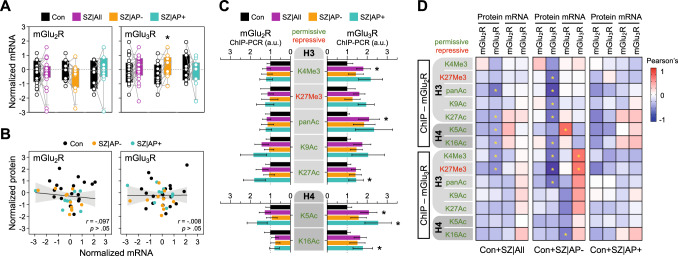
Gene expression and epigenetic regulation of mGlu_2_R and mGlu_3_R in the DLPFC of age-, sex- and PMI-matched pairs of schizophrenia (SZ) cases and controls, either altogether (All) or stratified by the absence (AP−) or presence (AP+) of antipsychotic drugs in the blood sample of the SZ subject pair at the time of death. **A** Box plots representing housekeeping gene-normalized levels of mGlu_2_R and mGlu_3_R mRNA (in arbitrary units [a.u.]). Paired *t*-tests detected significant differences (*p < 0.05) between the diagnosis groups for mGlu_3_R mRNA (AP-, *t*_1,11_ = 2.49)_,_ (**B**) Scatterplots depicting pairwise associations between mRNA levels and the corresponding immunodensities of mGlu_2_R or mGlu_3_R in the same DLPFC samples of control (Con) and SZ subjects. Fit line, as well as *r-* and *p*-values of the Pearson’s correlation test are shown for each analysis. **C** Bar plots representing the load of trimethylated (Me3) and/or acetylated (Ac; including pan-acetylated, panAc) lysine (K) residues of histones H3 (top plots) and H4 (bottom plots) attached to the promoter regions of the mGlu_2_R (left bars) or mGlu_3_R (right bars) coding genes (estimated by ChIP followed by qPCR assays [ChIP-PCR], and represented in percent from input values). Paired *t*-tests de*t*ected significant differences (*p < 0.05) between the diagnosis groups for the load of H3K4Me3 (All, *t*_1,18_ = 2.69), H3panAc (All, *t*_1,19_ = 2.13), H3K27Ac (AP + , *t*_1,9_ = 2.81), H4K5Ac (All_,_ *t*_1,18_ = 2.17; AP + , *t*_1,9 =_ 2.71), and H4K16Ac (AP + , *t*_1,9_ = 2.68) attached *t*o the mGlu_3_R (but not mGlu_2_R) gene. No overall similarities were observed between the load of HPTMs at the mGlu_2/3_R and GAPDH (used for housekeeping purposes [20]) genes, which may account for the specificity of the current results. **D** Heatmap representing pairwise associations between protein or mRNA levels of mGlu_2_R or mGlu_3_R (on the *x*-axis), and the amounts of HPTMs at histones H3 or H4 associated with the mGlu_2_R or mGlu_3_R gene promoters (on the *y*-axis) in the same DLPFC samples of control and SZ subjects altogether (Con+SZ|All), or stratified by the absence (Con+SZ|AP-) or presence (Con+SZ|AP+) of antipsychotic drugs. Color scale on the *r*-values is shown on the top-right corner. *FDR-adjusted *p* < 0.05.

Further exploratory assays were performed in DLPFC tissue samples from Cohort 1 subjects to address the possibility of an association between alterations in mGluR protein levels and dysregulated (epi)genetic mechanisms controlling the *GRM2* and/or *GRM3* genes. First, RT-qPCR assays were carried out with selective mGlu_2_R and mGlu_3_R mRNA probes (Supplemental Table S3) to estimate *GRM2* and *GRM3* gene expression, respectively (Fig. 4A). Despite the robust reduction of mGlu_2_R immunoreactivity in schizophrenia brains, no statistically significant differences in cortical mGlu_2_R mRNA expression were observed across the diagnostic groups and subgroups. Likewise, mGlu_3_R mRNA levels were similar in both schizophrenia cases (altogether) and controls. Surprisingly, AP-, but not AP+ schizophrenia subjects displayed greater mGlu_3_R mRNA levels (+64%, *p* < 0.05), as compared to matched controls (Fig. 4A). These observations largely contrasted with the above findings on mGlu_2/3_R immunodensities. Correlation analyses comparing mRNA and protein expression levels of these receptors in the same brain samples were not statistically significant (Fig. 4B).

ChIP analyses evaluated the amounts of key HPTMs at promoter-bound histones in the mGlu_2_R and mGlu_3_R coding genes. Consistent with mRNA expression levels, none of the permissive or repressive *GRM2*-associated HPTMs was altered in schizophrenia DLPFC samples (Fig. 4C). In contrast, schizophrenia samples displayed an overload of *GRM3*-associated HPTMs favoring gene expression, including H3K4Me3, H3panAc, H3K27Ac, H4K5Ac, and/or H4K16Ac (+61–110%, *p* < 0.05), with no alterations in the repressive mark studied (Fig. 4C). These alterations were mainly attributed to AP+ cases, especially histone H4-PTMs.

Overall, greater amounts of HPTMs in histones H3 and H4 at the mGlu_2_R and mGlu_3_R coding genes correlated with lower protein densities and/or greater mRNA levels of these receptors in postmortem human brain samples (Fig. 4D). These associations were more robust in case-control pairs that included the AP- subjects only (Fig. 4D, middle panel), in contrast with lack of correlation in the control-AP+ subgroup (Fig. 4D, right panel).

## Discussion

The present study explored potential alterations in molecular mechanisms regulating the expression of group II mGluRs in the DLPFC in schizophrenia, with a focus on associations with antipsychotic drugs detected in brain tissue. The main results indicate that schizophrenia is associated with downregulated protein expression of mGlu_2_R and mGlu_3_R, and antipsychotic medication may normalize mGlu_3_R, but not mGlu_2_R density. Regulation of mGlu_2_R and mGlu_3_R gene expression (i.e., mRNA levels) and epigenetic control (i.e., load of HPTMs at the mGlu_2/3_R gene promoters) did not parallel the observed protein alterations in the same brain samples, but rather showed opposite associations. Illness- and antipsychotic medication-associated alterations in mGlu_2_R (but not mGlu_3_R) densities were mimicked in rodent experiments.

**Fig. 5:**
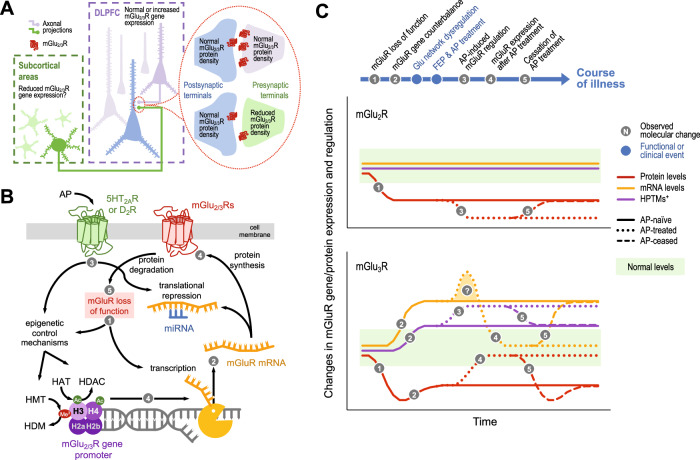
Graphical contextualization of the present data. **A** Cartoon illustrating a possible explanation of the observed changes in mGlu_2/3_R mRNA and protein cortical amounts from a neuroanatomical perspective. **B** Schematic flow diagram depicting the potential molecular mechanisms involved in the regulation of mGlu_2/3_R protein and mRNA expression in the DLPFC of schizophrenia subjects, and possible effects of antipsychotic (AP) medication on the regulatory pathway via 5HT_2A_R and/or D_2_R. Numbered circles correspond to the hypothetical, stepwise changes described in **C**. HAT histone acetyl transferase, HDAC histone deacetylase, HDM histone demethylase, HMT histone methyl transferase, miRNA microRNA. **C** Hypothetical model illustrating cortical changes in protein (red lines) and mRNA (yellow lines) expression, as well as epigenetic regulation at histone posttranslational modifications (PTMs; purple lines), of mGlu_2_R (top plot) and mGlu_3_R (bottom plot) over the course of schizophrenia development and illness progression, including effects of AP medication (dotted lines), following first-episode psychosis (FEP).

While the preferential presynaptic location of mGlu_2_R was expected, previous studies reported a postsynaptic location of mGlu_3_R in nonhuman primates and murine brains [33–36]. Recent data suggested a greater axonal (and presynaptic) location of mGlu_3_R in layers III and V of rat medial PFC [37]. Differences across species, or in antibody specificity may account for these contrasting reports. In the context of the high abundance of group II mGluRs at presynaptic terminals in human DLPFC, reconciling differences between protein densities and gene expression levels requires consideration of the different origins of neurons contributing to each measure. The presynaptic receptor proteins originate from both local neurons and anatomically distant neurons in subcortical or distributed cortical sites projecting to the DLPFC. In contrast, the mRNA is exclusively local in origin (Fig. 5A). Remarkably, thalamocortical projections exhibit extensive innervation of layer III within the DLPFC [38], wherein the most prominent punctate immunoreactivity of mGlu_3_R is localized [39, 40]. Stimulation of layer-III synaptic activity is indispensable to resolve working memory-dependent tasks [41], a cognitive domain largely impaired in schizophrenia patients. Of note, the sole study of mGlu_2_R and mGlu_3_R mRNA in thalamus showed no differences in expression between schizophrenia and control samples [42, 43] (see also Supplemental Table S1). Future immunohistochemical studies could evaluate the layer-specific manifestation of mGlu_2/3_R depletion in schizophrenia postmortem brains. This approach may elucidate the cellular origins of mGlu_2/3_R dysregulation and unraveling the clinical ramifications associated with their deficiency. Since mGlu_3_R is also expressed in astrocytes [44], we cannot discard that the observed alterations in schizophrenia samples may have a glial origin.

Alternatively, mRNA and protein level discordance may arise from compensatory feedback mechanisms. A hypothetical model describing changes in mGlu_2_R and mGlu_3_R gene and protein expression over the course of illness is illustrated in Fig. 5B, C. The model proposes that cortical densities of group II mGluRs are downregulated during neurodevelopment (Fig. 5B, C; Step 1). While the specific cause of mGlu_2/3_R downregulation in schizophrenia brains is unknown, the observation of lower mGlu_2_R densities in cortical samples from rodents prenatally exposed to poly(I:C) is consistent with a developmental origin (see also [45, 46]). mGlu_2/3_R deficiency may activate compensatory feedback mechanisms to counterbalance mGlu_2/3_R loss of function, which may involve increased mGlu_3_R gene expression, via modulation of the epigenetic control mechanisms (Fig. 5B, C; Step 2). However, this feedback mechanism may be insufficient to normalize mGlu_2/3_R protein levels, possibly explaining the inverse correlation between mGlu_3_R protein amounts and permissive HPTM loads at the mGlu_3_R promoter in the DLPFC of AP- schizophrenia samples. Prolonged mGlu_2/3_R loss of function may contribute to abnormal development of glutamatergic circuits, and the excitatory/inhibitory imbalance observed in patients [47]. Glutamatergic dysregulation, perhaps combined with other genetic and environmental risk factors, may trigger schizophrenia-related symptoms in early adulthood. Later, chronic antipsychotic treatment may further stimulate the epigenetic control mechanisms generating greater load of permissive HPTMs at the mGlu_3_R gene (Fig. 5B, C; Step 3). Consequently, mGlu_3_R protein density may return to normal values (Fig. 5B, C; Step 4), possibly contributing to the therapeutic action of antipsychotic drugs. Since we did not detect elevated mGlu_3_R transcript levels in AP+ subjects, the antipsychotic-induced stimulation of mGlu_3_R gene expression may only occur transiently, and mRNA levels may return to basal values once normal mGlu_3_R function is recovered. Alternatively, antipsychotic-induced inhibition of microRNAs repressing mGlu_3_R translation could explain the normalization of mGlu_3_R protein amounts in AP+ subjects [48, 49]. In contrast, antipsychotic medication (clozapine at least) may contribute to further downregulate mGlu_2_R cortical density. Since we cannot ignore the likelihood that AP- subjects were exposed to antipsychotic drugs earlier in their lives, it is possible that the prior mGlu_2/3_R imbalance is restored following treatment cessation (Fig. 5B, C; *Step 5*).

The model predicts schizophrenia DLPFC samples may display normal or low mGlu_2/3_R protein amounts, depending on the stage of illness at the time of death, and adherence with antipsychotic treatment. These effects, as well as those noted in the Introduction, may contribute to the large diversity of the reported results across the case-control studies analyzing mGlu_2/3_R in schizophrenia postmortem brains (Supplemental Table S1). Most of these studies did not report toxicological assessments, and the influence of antipsychotic drugs on the reported findings may have been overlooked. A recent postmortem brain study reporting toxicological assessments in a large schizophrenia case-control cohort did not find illness- or treatment-related alterations in mGlu_3_R immunodensities in the temporal lobe [31], indicating the brain region specificity of the abnormalities underlying schizophrenia.

A major goal of the present study was to evaluate the effect of antipsychotic treatment. Chronic blockade of 5HT_2A_R with clozapine was reported to reduce mGlu_2/3_R binding and mGlu_2_R mRNA levels via HDAC2 stimulation and selective hypoacetylation of the mGlu_2_R, but not mGlu_3_R gene promoter [7]. In contrast, our data suggests that in schizophrenia brains, antipsychotics may induce hyperacetylation of the mGlu_3_R, but not mGlu_2_R gene promoter and, consequently, increase mGlu_3_R immunodensity over that in AP- subjects. Since the vast majority of AP+ subjects were on SGAs proximate to death, no direct comparisons between FGA- and SGA-induced modulation of mGlu_2/3_R were possible. As alternatives, two complementary strategies were deployed. First, we compared mGlu_2/3_R cortical densities with predicted D_2_R and 5HT_2A_R occupancies. Greater 5HT_2A_R occupancy predicted higher densities of mGlu_3_R, but not mGlu_2_R, suggesting that stimulation of the epigenetic mechanisms facilitating mGlu_3_R gene expression may be associated with SGA-induced 5HT_2A_R blockade. The second approach evaluated mGlu_2/3_R cortical levels in rats treated with antipsychotics with low (i.e., haloperidol) or high (i.e., risperidone and clozapine) serotonergic/dopaminergic affinity ratios. In these experiments, chronic treatment with clozapine (but not haloperidol or risperidone) reduced the cortical density of mGlu_2_R, which may be compatible with the robust downregulation of this receptor in AP+ subjects. However, AP+ and AP- schizophrenia subgroups did not differ significantly in terms of their mGlu_2_R cortical densities. In turn, none of the antipsychotic treatments replicated the mGlu_3_R upregulation observed in schizophrenia AP+ subjects. Antipsychotic drug effects in schizophrenia patients with downregulated mGlu_2/3_R protein levels, may differ from those observed in ‘healthy’ rats. Differences in the brain regions studied (DLPFC versus whole cerebral cortex) may also explain the inconsistencies observed across species.

We also evaluated potential differences in the antipsychotic-induced regulation of mGlu_2/3_R in suicide and non-suicide schizophrenia cases. Arguably, subjects with schizophrenia who were on antipsychotic medication by the time of death and committed suicide would likely be classified as treatment-resistant patients. Since similar mGlu_2/3_R immunodensities were observed in suicide and non-suicide AP+ cases, regulation of these receptors may not be directly related to the efficacy of the antipsychotic treatment.

CB_1_R and D_2_R were evaluated to test the robustness of findings across studies. In agreement with our prior work [19], no changes were found in D_2_R between diagnostic groups. CB_1_R density was lower in AP+ schizophrenia brains, consistent with an overall consensus of downregulated CB_1_R expression in schizophrenia brains [50].

In conclusion, the present findings support a role for group II mGluRs in schizophrenia. Lower densities of mGlu_2/3_R may limit the efficacy of ligands targeting these receptors directly. The apparent normalization of mGlu_3_R by 5HT_2A_R/D_2_R antagonists may merit more investigation and consideration in clinical trial design, especially of mGlu_3_R agonists. Development of PET ligands for group II mGluRs may help resolve the time course of changes in the amounts of these receptors after initiation or withdrawal of antipsychotic treatment. Finally, development of larger samples of postmortem brain tissues from patients with schizophrenia may allow analyses of the effects of genetic variation on receptors, and provide a bridge to clinical trial design and interpretation.

### Supplementary information

Supplement 1
