# Supplementary material for: Effect of antipsychotic drugs on group II metabotropic glutamate receptor expression and epigenetic control in postmortem brains of schizophrenia subjects
Source: Transl Psychiatry. 2024 Feb 23;14:113. doi: 10.1038/s41398-024-02832-z (PMC10891050; doi:10.1038/s41398-024-02832-z)
Supplement: Supplementary file 1 — Supplement 1 [file 41398_2024_2832_MOESM1_ESM.pdf]

Supplement 1

Supplement to delaCuesta-Barrutia, *et al.*: Effect of antipsychotic drugs on group II metabotropic glutamate receptor expression and epigenetic control in postmortem brains of schizophrenia subjects

Table of Contents

SUPPLEMENTARY METHODS 2
Human brain specimens 2
Toxicological assessments in brain tissue and receptor occupancy estimates 3
Experimental animals 4
Antipsychotic drug treatments 5
Maternal immune activation model 5
Isolation and of pre- and postsynaptic terminals 6
Brain tissue homogenization for quantitative Western blotting 8
Antibodies 8
Standard SDS-PAGE and Western blotting 9
mRNA extraction and quantitative PCR. 10
Chromatin immunoprecipitation 11
SUPPLEMENTARY RESULTS 13
Validation of antibodies against GPCRs 13
Synaptic localization of $mGlu_{2/3}$, CB_1, and D_2 receptors 14
Effect of potentially confounding variables on GPCR immunodensities 15
SUPPLEMENTARY REFERENCES 16
SUPPLEMENTARY TABLES 19
Supplementary Table S1 19
Supplementary Table S2 21
Supplementary Table S3 23
Supplementary Table S4 24

Supplementary Table S5 ... *25*

Supplementary Table S6 ... *26*

Supplementary Table S7 ... *28*

SUPPLEMENTARY FIGURES AND LEGENDS ... 29

Supplementary Figure S1 ... *29*

Supplementary Figure S2 ... *30*

SUPPLEMENTARY METHODS

Human brain specimens

For the present study, we collected two case-control cohorts. Exploratory experiments were mainly performed in postmortem samples from Cohort 1 (n = 21 case-control pairs), collected between 2004-2010. Cohort 2 samples (n = 27 case-control pairs) were obtained between 2010-2018, and essentially used to confirm relevant findings in Cohort 1. Collective demographic and toxicological features of schizophrenia cases and controls in both cohorts are summarized in Table 1, and individual data are listed in Supplementary Table S2. Inclusion/exclusion criteria and sampling procedures were identical in both cohorts. During routine forensic investigations, retrospective search into individuals' medical histories first identified all schizophrenia cases. Only diagnoses performed by a board-certified psychiatrist of the Basque Healthcare System (*Osakidetza*) meeting DSM-IV or ICD-10 criteria were selected. Cases with other psychiatric (including alcohol/drug abuse disorders) or neurologic comorbidities were excluded from the study. Standard histopathologic assessments allowed to discard subjects with common age-related neuropathologies. For each schizophrenia case, a subject matching sex, age, and postmortem interval (PMI) was selected among the deceases occurred within a month, further controlling for storage time. Only subjects with available medical histories and no records of

severe illnesses, including mental and neurological disorders, were selected as controls. All deaths were sudden and unexpected, minimizing the agonal state, and thereby the impact of perimortem conditions on tissue quality and preservation.

Routine blood toxicological assessments were performed for all cases and controls during forensic investigations, which was annotated qualitatively, rather than quantitatively in most of the reports. Toxicological analyses screened for antipsychotic, antidepressant, and anxiolytic drugs, among many others. The presence of antipsychotic drugs in blood samples allowed to separate those schizophrenia cases who were on antipsychotic treatment (AP+) from those who were antipsychotic-free (AP-) at the time of death. AP- and AP+ schizophrenia subgroups did not differ significantly in terms of sex, age, and PMI (data not shown).

For quantitative neurochemical assays, grey matter samples from the dorsolateral prefrontal cortex (DLPFC), approximating Brodmann area 9, were carefully dissected following a standard human brain atlas [1]. To minimize the variability across subjects, white matter was carefully removed, and grey matter samples were immediately stored at -80ºC. Neuroimaging and postmortem brain studies suggest that DLPFC abnormalities may underlie schizophrenia-related impairment of executive functioning [2–5]. In parallel, toxicological assessments were performed in cerebellar and heart blood samples.

Toxicological assessments in brain tissue and receptor occupancy estimates

Liquid chromatography–tandem mass spectrometry (LC-MS/MS) was used as previously described [6] to quantify tissue concentrations of multiple psychoactive drugs (and/or active metabolites) in cerebellar postmortem specimens of 30 case-control pairs mainly from Cohort 2 (see Supplementary Table S2). Since the large fraction of drug bound to proteins (such as albumin) or lipids does not contribute to modify GPCRs activity, we first estimated the unbound

fraction (f_u), based on prior reports [e.g., see [7,8]]. Drug affinity (K_i) values for D_2R and $5HT_{2A}R$ of all drugs found in toxicological assessments were taken from the Plant-Derived Psychoactive Compounds database [9]. Since the database collects redundant information from multiple studies, we first filtered all those K_i values from assays using (*1*) the cloned human receptors with drugs of interest as test ligands, and (*2*) the same hot ligand as the competitor in the assays. The median from all filtered values was used as the K_i estimate in further occupancy calculations. The relative receptor occupancies of D_2R and $5HT_{2A}R$ were then estimated for each receptor-ligand pair according to the equation $FO = [L] / (K_i + [L])$ [10], where FO is the fractional occupancy, $[L]$ is the tissue nanomolar (nM) concentration of the assessed drug, and K_i is the drug's affinity constant for the corresponding receptor (in nM). In cases where more than one ligand for the assessed GPCR (D_2R or $5HT_{2A}R$) was present in the tissue, the largest occupancy value due to a single ligand was taken as the final estimate. Overall, transformation of drug concentrations into D_2R and $5HT_{2A}R$ relative occupancy values yielded reliable estimates, as many AP+ cases were within the range of receptor occupancy with expected therapeutic effects (i.e., 60-80%; see Figure 2C). Note, however, that some AP+ cases had such low concentrations of antipsychotic drugs that might have been considered AP-, at least in terms of D_2R occupancy.

Experimental animals

All procedures involving animal assays were previously approved by the Ethical Committee Board for Animal Research at the University of the Basque Country (UPV/EHU). Animals had free access to standard chow diet and water, and were housed under controlled conditions of temperature and humidity, with a 12/12-h light/dark cycle. C57BL/6J mice and Sprague-Dawley rats were originally purchased at Envigo (Barcelona, Spain). The $mGlu_2R$-deficient (*Grm2*$^{-/-}$) mouse line was a generous gift from Dr. Javier Gonzalez-Maeso (The Virginia Commonwealth

University, VA USA), and bred in UPV/EHU animal facilities. Brain samples from CB_1R knockout (*Cnr1*$^{-/-}$) mice and wild-type littermates were kindly donated by Dr. Giovanni Marsicano (Université de Bordeaux, France). Generation and genotyping of *Grm2*$^{-/-}$ [11] and *Cnr1*$^{-/-}$ [12] mice was described elsewhere.

Antipsychotic drug treatments

Adult male Sprague-Dawley rats (300-350 g at the onset of the experiments) were treated intraperitoneally (i.p.) with saline (SAL; 1 ml/kg), haloperidol (HAL; 0.5 mg/kg), risperidone (RIS; 0.5 mg/kg), or clozapine (CLO; 5 mg/kg) every 12 h for 21 consecutive days, as described elsewhere [13]. Rats were killed by decapitation 48 hours after the last injection of clozapine and risperidone, or 72 hours after the last injection of haloperidol and saline. The above doses were reported to yield serum concentrations comparable to those observed in patients receiving these treatments, and washout time intervals were adjusted according to the pharmacokinetic properties of each drug [14]. Brains were removed and whole cerebral cortices were dissected on ice and stored at -80ºC until the assays.

Maternal immune activation model

Viral infections during pregnancy, as well as other obstetric complications boosting maternal immune activation (MIA), are associated with increased risk of schizophrenia (and other mental illnesses) in the offspring [15]. Indeed, murine MIA models recapitulate some of the core symptoms of the schizophrenia syndrome, and display translational validity [15,16]. A standardized protocol [17,18] was used to reproduce the MIA model in C57BL/6J mice with the polyinosinic-polycytidylic acid [poly(I:C), or PIC; Sigma-Aldrich, Saint Louis MO, USA], an analogue of the double-stranded (ds) RNA, stimulating Toll-like receptor 3 [16]. Briefly, pregnant dams (n = 2) received a single dose of poly(I:C) (5 mg/kg, i.p.) at gestational day (GD) 9.5. In parallel, control

mothers (n = 2) received vehicle solution (0.9% NaCl; 10 ml/kg) on the same GD. The selected poly(I:C) dose was previously shown to elicit a robust immune response in pregnant dams, and a schizophrenia-like behavioral phenotype in the offspring under our experimental conditions [17–19]. Dams and the offspring were left undisturbed until weaning (postnatal day [PND] 21). On PND 84, mice prenatally exposed to poly(I:C) (n = 3 females + 4 males) or saline (n = 4 females + 5 males) were killed by decapitation, and the cerebral cortices were dissected on ice and stored at -80ºC until the assays.

Isolation and of pre- and postsynaptic terminals

To characterize the pre- and/or postsynaptic localization of the studied GPCRs using KO-validated antibodies, cortical samples from three neurotypic control subjects (all males, 35-45 years old, 7-15 h of PMI) were fractionated by sequential (ultra)centrifugation steps as indicated previously [20]. Briefly, one gram of carefully dissected grey matter tissue from the DLPFC was homogenized in 30 mL of ice-cold Tris-sucrose buffer, containing 5 mM Tris-HCl, pH 7.4, 0.32 M sucrose, 5 mM Na_3VO_4, 10 mM NaF, and 50 µL/g of a commercial protease inhibitor cocktail (Sigma-Aldrich), using a motorized glass/Teflon Potter Elvehjem tissue grinder. The clearance between the Teflon pestle and the glass tube ranged 0.13–0.18 µm, which is known to maximize the encapsulation of the synaptic terminals, and subsequent synaptosome formation, during the grinding of the tissue [21]. To remove the nuclear fraction, along with cell and tissue debris, crude brain homogenates were centrifuged in a 4ºC-refrigerated Sorvall RC-5C centrifuge, equipped with a SM-24 rotor, at 1,100×g for 15 min. Cell membranes and membranous organelles were then precipitated from the supernatants at 40,000×g for 10 min. The pellets were washed in ice-cold Tris-sucrose buffer, recentrifuged under the above conditions, and resuspended in 3 mL of ice-cold Tris-sucrose buffer. The resuspended membranes were gently overlaid onto a

discontinuous sucrose gradient consisting of 3 mL of each 1.2 (bottom), 1.0 (middle), and 0.85 M (top) sucrose solutions, all buffered with 5 mM Tris HCl pH 7.4, and supplemented with the above protease and phosphatase inhibitors. The samples were centrifuged for 2 h at 100,000×*g* in a refrigerated XE-90 ultracentrifuge (Beckman Coulter, Brea CA, USA), equipped with a SW 32.1 swinging bucket rotor. The interface formed between the 1.0 and 1.2 M sucrose solutions, containing the synaptosomal fraction, was immediately collected and diluted in 8 mL of washing buffer (5 mM Tris-HCl pH 7.4, supplemented with protease and phosphatase inhibitors), and recentrifuged at 40,000×g for 10 min at 4ºC. To fully eliminate sucrose, the resulting pellets were resuspended in 1 mL of washing buffer, and recentrifuged at 21,000×*g* for 15 min. The supernatants were discarded and the clean synaptosomal fractions were incubated for 60 min, at 4ºC, with gentle rotation in 5 mL of dissociation buffer, containing 20 mM Tris-HCl pH 8.0, 1% Triton X-100, and the protease and phosphatase inhibitor cocktails, which disrupts the bonds between the pre- and postsynaptic membranes. Samples were then ultracentrifuged (140,000×*g*, 30 min, 4ºC), and the supernatants containing the presynaptic membrane fraction were carefully collected and stored at -80ºC. The final pellets, corresponding to the postsynaptic density (PSD) fraction, were resuspended in 900 μL of 50 mM Tris-HCl pH 7.4 buffer, aliquoted and stored at -80ºC until the assays. Aliquots from the pre- and postsynaptic fractions were separated during the assays to estimate the total protein concentrations by the DC Protein Assay method (Bio-Rad, Hercules CA, USA). Before Western blot analyses (see below), pre- and postsynaptic synaptosomal extractions were mixed with equal volumes of Laemmli buffer 2× (100 mM Tris-HCl, pH 6.8, 20% glycerol, 4% sodium dodecyl sulfate [SDS], 5% β-mercaptoethanol, 0.02% bromophenol blue),

Brain tissue homogenization for quantitative Western blotting

Cohort 1 specimens, as well as rodent cortical tissues, were prepared as total homogenates for multitarget assessments. Briefly, ~200-mg DLPFC tissue samples were homogenized with a T25 Ultraturrax (IKA, Staufen, Germany) in phosphate-buffered saline (PBS, pH 7.4) supplemented with 1% of protease and phosphatase inhibitor cocktails (Sigma-Aldrich). Protein concentration was quantified in each brain homogenate with the DC assay (Bio-Rad). Appropriate volumes of homogenization buffer were then added to equalize protein concentrations across samples. Finally, brain homogenates were mixed with equal volumes of the above Laemmli buffer 2×, aliquoted, and stored at -80ºC.

Since Cohort 2 was specifically aimed at quantifying cortical immunodensities of target GPCRs, whole membrane extractions were prepared from tissue samples. Frozen DLPFC specimens of about 300 mg were homogenized in 3 mL PBS containing 0.32 M sucrose, and 1% protease and phosphatase inhibitors. Nuclei and tissue debris were removed by centrifugation at 1,100×*g* for 10 min at 4ºC. Supernatants were again centrifuged (35,000×*g*, 15 min, 4ºC) to precipitate cell membranes. Pellets were washed twice in equal volumes of washing buffer (PBS supplemented with protease and phosphatase inhibitors), and recentrifuged (35,000×g, 15 min, 4ºC). Pellets were then resuspended in 1 mL of washing buffer, and protein concentrations were estimated as above. Samples were then divided into 0.5-mg protein aliquots, and stored as pellets at -80ºC following centrifugation (35,000×g, 15 min, 4ºC). Before quantitative assays, samples were resuspended in 250 µl of washing buffer, and mixed with 250 µl of Laemmli buffer 2×.

Antibodies

A detailed list of primary antibodies used in the study is provided in Supplementary Table S3. Commercial antibodies against $mGlu_2$, $mGlu_3$, CB_1, and D_2 receptors were all validated in

genetically modified mice lacking the target receptor, and further characterized herein. Peroxidase-conjugated secondary antibodies against mouse or rabbit IgG+M were from Jackson ImmunoResearch Laboratories (West Grove, PA, USA) or Invitrogen (Waltham, MA, USA).

Standard SDS-PAGE and Western blotting

Prior to quantitative experiments, a standard sample was prepared by pooling equal amounts of all control samples, and fractionated in working aliquots. Standard samples were loaded in triplicate in all experimental gels to compare results across the membranes (see below). For quantitative analyses, samples were first denatured at 70ºC for 5 min and 10- or 20-μg protein aliquots loaded into 10% SDS-polyacrylamide gels (SDS-PAGE). These amounts were previously determined to be within the linear range for densitometric quantifications (data not shown). After electrophoresis, proteins were transferred to nitrocellulose membranes, and subsequently blocked (1 h at room temperature), and incubated with primary (overnight, 4ºC; Supplementary Table S3) and secondary (1 h at room temperature; 1:5,000) antibodies, in PBS supplemented with 5% skim milk and 0.1% Tween-20. Chemiluminescence was induced with commercial ECL reagents (Thermo Scientific Pierce; Waltham, MA, USA), and images were digitized using an AI680 Imager (Cytiva, Marlborough, MA, USA). Membranes were finally stripped and reprobed with antibodies targeting β-actin, allowing data normalization. Image Studio™ Lite v5.2 (Li-COR; Lincoln, NE, USA) was used for densitometric quantification. To calculate the immunoreactivity of a given lane in a gel, the raw integrated optical density (IOD) was first obtained by outlining the stained area manually with the rectangle tool. The 'rolling ball' algorithm was used to subtract background, as estimated from the top and bottom edges of the rectangle. This approach accounts for both the size and the intensity of the band analyzed, and accurately removes background noise. The manual selection process was repeated for all

samples in a gel. Every sample was assessed in three different gels, and the IOD was calculated in percent to a standard sample loaded in triplicate in all gels. This procedure was reported to reduce variability between subjects [22,23]. For synaptosomal extractions, the IODs from a target protein were obtained as indicated above, and the percent distribution was estimated for each subject as the IOD in the pre- or postsynaptic fraction divided by the sum of the IODs in both fractions. The mean (and standard error) of the IODs in one fraction across all three subjects was estimated and plotted.

mRNA extraction and quantitative PCR.

Expression levels of $mGlu_2R$ and $mGlu_3R$ mRNA were quantified by real-time quantitative PCR (RT-qPCR) analyses in human postmortem brain tissue samples, as previously described [24]. Briefly, grey matter tissue samples from the DLPFC were processed with RiboPureTM extraction kit (ThermoFisher, Waltham, MA, USA) to purify whole sample RNA. Concentration of total RNA in the sample was estimated in a NanoDrop 1000 Spectrophotometer (ThermoFisher). To approach RNA extraction quality, the RNA integrity number (RIN) was estimated using Agilent RNA 6000 Nano kit in a 2100 Bioanalyzer (Agilent, Santa Clara, CA, USA). Following DNase digestion with Deoxyribonuclease I (Thermo Fisher), 1-µg sample aliquots of total RNA were converted into single-stranded cDNA using High-Capacity cDNA Reverse Transcription kit (ThermoFisher), following manufacturer instructions. Quantitative real time PCR (qPCR) was performed on a StepOneTM system (Thermo Fisher), with Fast SYBR Green Master Mix (ThermoFisher). Reactions were loaded with 40 ng of total cDNA, along with the forward and reverse primers corresponding to the target gene (Supplementary Table S4), in a final volume of 10 µl. At the end of each run, dissociation curves were analyzed for quality control. mRNA expression of GAPDH and RPS13 housekeeping genes as well as that of a

reference sample (pool of control samples) was used to normalize the mRNA expression levels of all other reported genes using ΔΔCt method: $\Delta\Delta Ct = (Ct\ (\text{target gene})_{sample} - Ct\ (\text{reference gene})_{sample}) - (Ct\ (\text{target gene})_{reference\ sample} - Ct\ (\text{reference gene})_{reference\ sample})$. Samples were run in triplicate, and appropriate positive and negative controls were included in all the experiments.

Chromatin immunoprecipitation

Chromatin immunoprecipitation (ChIP) was essentially achieved as described elsewhere [24]. Approximately 120-mg samples of the grey matter were dissected from postmortem human DLPFC tissues, and quickly homogenized in ice-cold 10 mM Tris-HCl buffer pH 7.5, supplemented with 4 mM $MgCl_2$, 1 mM $CaCl_2$ followed by incubation with 1 U/ml of micrococcal nuclease (MNase; Sigma-Aldrich) at 37°C for 10 min. MNase activity was stopped by adding EDTA to a final concentration of 10 mM. Samples were mixed with a hypotonic lysis solution, containing 0.1 mM benzamidine, 0.1 mM phenylmethylsulfonylfluoride (PMSF), 1.5 mM 1,4-dithiothreitol (DTT), and 0.2 mM EDTA, and incubated on ice for 1 h. Insoluble tissue debris were removed by centrifugation (1,000×*g*, 10 min, 4°C), and the supernatants, containing soluble chromatin, were mixed with 10× incubation buffer (200 mM Tris-HCl, pH 7.5, 50 mM EDTA, 500 mM NaCl). 60 µl of diluted chromatin (input sample) were removed and saved at 4 °C until elution. For immunoprecipitation (IP), primary antibodies against one of the following histone H3 or H4 posttranslational modifications (HPTMs): H3K4Me3, H3K27Me3, H3panAc, H3K9Ac, H3K27Ac, H4K5Ac, H4K16Ac (see Supplementary Table S5 for further details) and 20 µl of fully suspended protein A-coated magnetic beads (Millipore, Burlington, MA, USA) were added to aliquots of diluted chromatin and incubated overnight at 4 °C with rotation. The magnetic beads were separated along with the IP products by placing the reaction tubes on a magnet and removing the supernatants. Next, pellets were sequentially washed in the following

ice-cold buffers: low (20 mM Tris-HCl pH 8.1, 150 mM NaCl, 2 mM EDTA, 0.1% SDS, 1% Triton X-10) and high (20 mM Tris-HCl pH 8.1, 500 mM NaCl, 2 mM EDTA, 0.1% SDS, 1% Triton X-100) salt buffers; LiCl immune complex wash buffer (10 mM Tris-HCl pH 8.1, 250 mM LiCl, 1 mM EDTA, 0.01% IGEPAL CA630, 0.01% deoxycholic acid) and TE buffer (10 mM Tris-HCl pH 8.1, 1 mM EDTA,). Finally, the IP products were eluted by incubation in ChIP elution buffer (1% SDS, 0.1 M $NaHCO_3$) with proteinase K (100 ug/ml) at 62 °C for 2 h with shaking followed by a 10 min incubation at 95 °C. Magnetic beads were separated from antibody-chromatin complex, and the DNA in the samples (including the input) was purified with QIAquick PCR purification kit (Qiagen, Hilden, Germany) following the manufacturer's instructions. Final DNA volume was 1:4 diluted in molecular grade water.

The promoter regions of the $mGlu_2R$ and $mGlu_3R$ coding genes were amplified in each IP reaction for subject using selective forward and reverse primers (Supplementary Table S4). Fold changes relative to 6% of input DNA were determined using the comparative Ct method, where $\Delta Ct = Ct\ (\text{target gene})_{\text{immunoprecipitated DNA sample}} - Ct\ (\text{target gene})_{\text{6\% input DNA}}$. The relative amounts of immunoprecipitated DNA were calculated as $2^{-\Delta Ct}$ values, which are proportional to the load of a particular HPTM in the promoter regions of the target genes.

SUPPLEMENTARY RESULTS

Validation of antibodies against GPCRs

All antibodies used in quantitative immunoblotting experiments were previously validated in brain samples from knockout animals lacking the target receptor. Up to four different antibodies raised against $mGlu_2R$ were tested (Supplementary Table S4). Only two (ab15672 [now discontinued] and AB-N32) fulfilled validation criteria: (1) detection of a band that matches the expected molecular weight of the receptor (i.e., ~95 kDa for $mGlu_2R$) in both human and wild-type (WT) murine brain samples, and (2) no band detection at the same molecular size in $mGlu_2R^{-/-}$ mice (Figure 1A; Supplementary Figure S1A). Similarly, the anti-CB_1R antibody selected for quantitative studies (ab23703) recognized a ~50-kDa band in human and WT mouse brains, which was absent in $CB_1R^{-/-}$ animals (Figure 1A; Supplementary Figure S1B). Other bands immunodetected by this antibody, as well as other anti-CB_1R antibodies tested here were considered unspecific. Other tested anti-CB_1R antibodies (i.e., ImmunnoGenes) showed inconsistent results across batches (Supplementary Figure S1B), and were discarded for the present study. The selectivity of both anti-$mGlu_3R$ (ab166608) and anti-D_2R (AB5084P) antibodies was previously demonstrated using respective target knockout animals [25,26]. The recognition patterns under the current experimental conditions were similar to those in prior studies (Figure 1A). Thus, in human and mouse brains, monomeric $mGlu_3R$ was detected at ~110 kDa along with a secondary immunoreactive band at ~250 kDa, presumably corresponding to receptor oligomers [26]. On the other hand, anti-D_2R antibody consistently reacted against two receptor species in human mouse cortex: a major ~75 kDa band, and a putatively glycosylated receptor form of ~100 kDa. While we focused in major ~110-kDa $mGlu_3R$ and ~75-kDa D_2R species, other receptor forms (referred to as $mGlu_3R^{olig}$ and D_2R^{100k}) were also quantified.

Synaptic localization of $mGlu_{2/3}$, CB_1, and D_2 receptors

The purity of the synaptic extractions was first evaluated by Western blotting with specific markers for the pre-[synaptophysin (SYP) and synaptosome-associated protein of 25 kDa (SNAP25)] and post-(PSD95) synaptic terminals (Figure 1B). As expected, in all three tested subjects, the presynaptic protein extraction had negligible PSD95 immunoreactivity, as compared to that in the postsynaptic fraction, whereas SYP was essentially absent from the postsynaptic fractions, compared to the amounts observed in the presynaptic extractions. While the vast majority of SNAP25 immunoreactivity was observed in the presynaptic terminals, small amounts were observed in the postsynaptic fractions, as SNARE proteins play a role in postsynaptic membrane protein trafficking [27]. In turn, similar amounts of β-actin were found at both synaptic endings. These observations indicate a large purity of the pre- and postsynaptic protein extractions.

$mGlu_2R$ and $mGlu_3R$ were immunodetected at both terminals, although greater presynaptic (Pre: 73-75%) than postsynaptic (Post: 25-27%) abundancies were observed in all three subjects (Figure 1B). More selective presynaptic localization was found for $mGlu_3R^{olig}$ (Pre: 88%; Post: 12%), and a similar distribution was observed for CB_1R (Pre: 79%; Post: 21%). Surprisingly, the two D_2R species studied were only immunodetected in the postsynaptic extractions (Pre: 4%; Post: 96%). However, a ~150 kDa band in the D_2R immunoblot (pointed with an asterisk in Figure 1B) was mainly observed in the presynaptic terminal, suggesting that D_2R may be subjected to different posttranslational modifications at both sides of the synaptic cleft that alter significantly its molecular weight. Unfortunately, this putative presynaptic D_2R species was barely detected in total homogenates, and no further evaluations were made regarding this receptor form.

Effect of potentially confounding variables on GPCR immunodensities

In the overall sample, longer postmortem interval (PMI) was associated with lower cortical immunoreactivity of $mGlu_3R$ oligomers (r = -0.293, $P < 0.01$) and D_2R monomer (r = -0.341, $P < 0.001$) (Figure 1C). Men displayed slightly greater amounts of $mGlu_3R^{olig}$ species than women (+23%, $P < 0.05$), while aging contributed to increase D_2R immunoreactivity (r = 0.230, $P < 0.05$), and perhaps decrease that of $mGlu_3R$ in control subjects (r = 0.310, $P < 0.05$). Storage time and brain pH had no significant impact on receptor immunodensities. The potential influence of psychoactive drugs on GPCR brain amounts was evaluated separately in the diagnosis groups, as cases and controls displayed categorical differences in their toxicological profiles. Interestingly, in subjects with schizophrenia, antipsychotic drugs altered mGluRs in opposite directions, slightly reducing mGu_2R (-15%, $P > 0.05$), while increasing mGu_3R (+49%, $P < 0.001$) cortical immunodensities (Figure 1C). In controls, benzodiazepine medication appeared to downregulate most of the GPCRs evaluated. Of note, only 3 in 48 individuals in the control group had detectable concentrations of drugs in this category (see Table 1 and Supplementary Table S2), and therefore, the contribution of these drugs to the overall observations of the study may be marginal. Besides, these 3 subjects had significantly longer PMI values (+15.4 h, $P < 0.001$). Consequently, parallel analyses to discard any potential bias in the results showed that all benzodiazepine effects on GPCR densities vanished when adjusting by PMI (data not shown). No other associations between the studied GPCR brain amounts and the potentially confounding variables were found.

SUPPLEMENTARY REFERENCES

1 Mai JK, Assheuer J, Paxinos G. *Atlas of the human brain*. 3rd ed. Academic Press: San Diego, CA, 1997.

2 Callicott JH, Bertolino A, Mattay VS, Langheim FJP, Duyn J, Coppola R *et al.* Physiological Dysfunction of the Dorsolateral Prefrontal Cortex in Schizophrenia Revisited. *Cereb Cortex* 2000; **10**: 1078–1092.

3 Fuster JM. *The Prefrontal Cortex: Anatomy, Physiology, and Neuropsychology of the Frontal Lobe*. 3rd Ed. Lippincott-Raven: Philadelphia, 1997.

4 Hoftman GD, Datta D, Lewis DA. Layer 3 Excitatory and Inhibitory Circuitry in the Prefrontal Cortex: Developmental Trajectories and Alterations in Schizophrenia. *Biol Psychiatry* 2017; **81**: 862–873.

5 Glausier JR, Lewis DA. Mapping pathologic circuitry in schizophrenia. *Handb Clin Neurol* 2018; **150**: 389–417.

6 Sampedro MC, Unceta N, Gómez-Caballero A, Callado LF, Morentin B, Goicolea MA *et al.* Screening and quantification of antipsychotic drugs in human brain tissue by liquid chromatography-tandem mass spectrometry: application to postmortem diagnostics of forensic interest. *Forensic Sci Int* 2012; **219**: 172–178.

7 Liu X, Van Natta K, Yeo H, Vilenski O, Weller PE, Worboys PD *et al.* Unbound drug concentration in brain homogenate and cerebral spinal fluid at steady state as a surrogate for unbound concentration in brain interstitial fluid. *Drug Metab Dispos* 2009; **37**: 787–793.

8 Musteata FM. Calculation of normalized drug concentrations in the presence of altered plasma protein binding. *Clin Pharmacokinet* 2012; **51**: 55–68.

9 Roth BL, Lopez E, Beischel S, Westkaemper RB, Evans JM. Screening the receptorome to discover the molecular targets for plant-derived psychoactive compounds: a novel approach for CNS drug discovery. *Pharmacol Ther* 2004; **102**: 99–110.

10 Rivero G, Llorente J, McPherson J, Cooke A, Mundell SJ, McArdle CA *et al.* Endomorphin-2: a biased agonist at the μ-opioid receptor. *Mol Pharmacol* 2012; **82**: 178–188.

11 Yokoi M, Kobayashi K, Manabe T, Takahashi T, Sakaguchi I, Katsuura G *et al.* Impairment of hippocampal mossy fiber LTD in mice lacking mGluR2. *Science (80-)* 1996; **273**: 645–647.

12 Marsicano G, Wotjak CT, Azad SC, Bisogno T, Rammes G, Cascio MG *et al.* The endogenous cannabinoid system controls extinction of aversive memories. *Nature* 2002; **418**: 530–534.

13 Barakauskas VE, Beasley CL, Barr AM, Ypsilanti AR, Li H-Y, Thornton AE *et al.* A novel mechanism and treatment target for presynaptic abnormalities in specific striatal regions in schizophrenia. *Neuropsychopharmacology* 2010; **35**: 1226–1238.

14 Kapur S, VanderSpek SC, Brownlee BA, Nobrega JN. Antipsychotic dosing in preclinical models is often unrepresentative of the clinical condition: a suggested solution based on in vivo occupancy. *J Pharmacol Exp Ther* 2003; **305**: 625–631.

15 Meyer U. Prenatal Poly(I:C) exposure and other developmental immune activation models in rodent systems. *Biol Psychiatry* 2014; **75**: 307–315.

16 Reisinger S, Khan D, Kong E, Berger A, Pollak A, Pollak DD. The poly(I:C)-induced maternal immune activation model in preclinical neuropsychiatric drug discovery. *Pharmacol Ther* 2015; **149**: 213–226.

17 Prades R, Munarriz-Cuezva E, Urigüen L, Gil-Pisa I, Gómez L, Mendieta L *et al.* The prolyl oligopeptidase inhibitor IPR19 ameliorates cognitive deficits in mouse models of schizophrenia. *Eur Neuropsychopharmacol* 2017; **27**: 180–191.

18 MacDowell KS, Munarriz-Cuezva E, Caso JR, Madrigal JLM, Zabala A, Meana JJ *et al.* Paliperidone reverts Toll-like receptor 3 signaling pathway activation and cognitive deficits in a maternal immune activation mouse model of schizophrenia. *Neuropharmacology* 2017; **116**: 196–207.

19 García-Bueno B, Gassó P, MacDowell KS, Callado LF, Mas S, Bernardo M *et al.* Evidence of activation of the Toll-like receptor-4 proinflammatory pathway in patients with schizophrenia. *J Psychiatry Neurosci* 2016; **41**: E46-55.

20 Erdozain AM, Brocos-Mosquera I, Gabilondo AM, Meana JJ, Callado LF. Differential α(2A)- and α(2C)-adrenoceptor protein expression in presynaptic and postsynaptic density fractions of postmortem human

prefrontal cortex. *J Psychopharmacol* 2019; **33**: 244–249.

21 Gray EG, Whittaker VP. The isolation of nerve endings from brain: an electron-microscopic study of cell fragments derived by homogenization and centrifugation. *J Anat* 1962; **96**: 79–88.

22 Gil-Pisa I, Munarriz-Cuezva E, Ramos-Miguel A, Uriguen L, Meana JJ, Garcia-Sevilla JA. Regulation of munc18-1 and syntaxin-1A interactive partners in schizophrenia prefrontal cortex: down-regulation of munc18-1a isoform and 75 kDa SNARE complex after antipsychotic treatment. *Int J Neuropsychopharmacol* 2012; **15**: 573–588.

23 McCullumsmith RE, Meador-Woodruff JH. Novel approaches to the study of postmortem brain in psychiatric illness: old limitations and new challenges. *Biol Psychiatry* 2011; **69**: 127–133.

24 Brocos-Mosquera I, Miranda-Azpiazu P, Muguruza C, Corzo-Monje V, Morentin B, Meana JJ *et al.* Differential brain ADRA2A and ADRA2C gene expression and epigenetic regulation in schizophrenia. Effect of antipsychotic drug treatment. *Transl Psychiatry* 2021; **11**: 643.

25 Stojanovic T, Orlova M, Sialana FJ, Höger H, Stuchlik S, Milenkovic I *et al.* Validation of dopamine receptor DRD1 and DRD2 antibodies using receptor deficient mice. *Amino Acids* 2017; **49**: 1101–1109.

26 García-Bea A, Walker MA, Hyde TM, Kleinman JE, Harrison PJ, Lane TA. Metabotropic glutamate receptor 3 (mGlu3; mGluR3; GRM3) in schizophrenia: Antibody characterisation and a semi-quantitative western blot study. *Schizophr Res* 2016; **177**: 18–27.

27 Antonucci F, Corradini I, Fossati G, Tomasoni R, Menna E, Matteoli M. SNAP-25, a Known Presynaptic Protein with Emerging Postsynaptic Functions. *Front Synaptic Neurosci* 2016; **8**: 7.

28 Ohnuma T, Augood SJ, Arai H, McKenna PJ, Emson PC. Expression of the human excitatory amino acid transporter 2 and metabotropic glutamate receptors 3 and 5 in the prefrontal cortex from normal individuals and patients with schizophrenia. *Mol Brain Res* 1998; **56**: 207–217.

29 Richardson-Burns SM, Haroutunian V, Davis KL, Watson SJ, Meador-Woodruff JH. Metabotropic glutamate receptor mRNA expression in the schizophrenic thalamus. *Biol Psychiatry* 2000; **47**: 22–28.

30 Crook JM, Akil M, Law BCW, Hyde TM, Kleinman JE. Comparative analysis of group II metabotropic glutamate receptor immunoreactivity in Brodmann's area 46 of the dorsolateral prefrontal cortex from patients with schizophrenia and normal subjects. *Mol Psychiatry* 2002; **7**: 157–164.

31 Gupta DS, McCullumsmith RE, Beneyto M, Haroutunian V, Davis KL, Meador-Woodruff JH. Metabotropic glutamate receptor protein expression in the prefrontal cortex and striatum in schizophrenia. *Synapse* 2005; **57**: 123–131.

32 Corti C, Crepaldi L, Mion S, Roth AL, Xuereb JH, Ferraguti F. Altered Dimerization of Metabotropic Glutamate Receptor 3 in Schizophrenia. *Biol Psychiatry* 2007; **62**: 747–755.

33 Tkachev D, Mimmack ML, Huffaker SJ, Ryan M, Bahn S. Further evidence for altered myelin biosynthesis and glutamatergic dysfunction in schizophrenia. *Int J Neuropsychopharmacol* 2007; **10**: 557–563.

34 Gonzalez-Maeso J, Ang RL, Yuen T, Chan P, Weisstaub N V, Lopez-Gimenez JF *et al.* Identification of a serotonin/glutamate receptor complex implicated in psychosis. *Nature* 2008; **452**: 93–97.

35 Bullock WM, Cardon K, Bustillo J, Roberts RC, Perrone-Bizzozero NI. Altered expression of genes involved in GABAergic transmission and neuromodulation of granule cell activity in the cerebellum of schizophrenia patients. *Am J Psychiatry* 2008; **165**: 1594–1603.

36 Ghose S, Crook JM, Bartus CL, Sherman TG, Herman MM, Hyde TM *et al.* Metabotropic Glutamate Receptor 2 and 3 Gene Expression in The Human Prefrontal Cortex and Mesencephalon in Schizophrenia. *Int J Neurosci* 2008; **118**: 1609–1627.

37 Sartorius LJ, Weinberger DR, Hyde TM, Harrison PJ, Kleinman JE, Lipska BK. Expression of a GRM3 splice variant is increased in the dorsolateral prefrontal cortex of individuals carrying a schizophrenia risk SNP. *Neuropsychopharmacol Off Publ Am Coll Neuropsychopharmacol* 2008; **33**: 2626–2634.

38 Ghose S, Gleason KA, Potts BW, Lewis-Amezcua K, Tamminga CA. Differential expression of metabotropic glutamate receptors 2 and 3 in schizophrenia: a mechanism for antipsychotic drug action? *Am J Psychiatry* 2009; **166**: 812–820.

39 Ghose S, Chin R, Gallegos A, Roberts R, Coyle J, Tamminga C. Localization of NAAG-related gene expression deficits to the anterior hippocampus in schizophrenia. *Schizophr Res* 2009; **111**: 131–137.

40 Frank E, Newell KA, Huang X-F. Density of metabotropic glutamate receptors 2 and 3 (mGluR2/3) in the

dorsolateral prefrontal cortex does not differ with schizophrenia diagnosis but decreases with age. *Schizophr Res* 2011; **128**: 56–60.

41 Matosin N, Fernandez-Enright F, Frank E, Deng C, Wong J, Huang X-F *et al.* Metabotropic glutamate receptor mGluR2/3 and mGluR5 binding in the anterior cingulate cortex in psychotic and nonpsychotic depression, bipolar disorder and schizophrenia: implications for novel mGluR-based therapeutics. *J Psychiatry Neurosci* 2014; **39**: 407–416.

42 Kim S, Zavitsanou K, Gurguis G, Webster MJ. Neuropathology markers and pathways associated with molecular targets for antipsychotic drugs in postmortem brain tissues: exploration of drug targets through the Stanley Neuropathology Integrative Database. *Eur Neuropsychopharmacol J Eur Coll Neuropsychopharmacol* 2012; **22**: 683–694.

43 Moreno JL, Miranda-Azpiazu P, Garcia-Bea A, Younkin J, Cui M, Kozlenkov A *et al.* Allosteric signaling through an mGlu2 and 5-HT2A heteromeric receptor complex and its potential contribution to schizophrenia. *Sci Signal* 2016; **9**: ra5.

44 McOmish CE, Pavey G, Gibbons A, Hopper S, Udawela M, Scarr E *et al.* Lower [3H]LY341495 binding to mGlu2/3 receptors in the anterior cingulate of subjects with major depressive disorder but not bipolar disorder or schizophrenia. *J Affect Disord* 2016; **190**: 241–248.

45 Dean B, Duncan C, Gibbons A. Changes in levels of cortical metabotropic glutamate 2 receptors with gender and suicide but not psychiatric diagnoses. *J Affect Disord* 2019; **244**: 80–84.

46 Neki A, Ohishi H, Kaneko T, Shigemoto R, Nakanishi S, Mizuno N. Pre- and postsynaptic localization of a metabotropic glutamate receptor, mGluR2, in the rat brain: an immunohistochemical study with a monoclonal antibody. *Neurosci Lett* 1996; **202**: 197–200.

47 Moreno JL, Muguruza C, Umali A, Mortillo S, Holloway T, Pilar-Cuellar F *et al.* Identification of three residues essential for 5-hydroxytryptamine 2A-metabotropic glutamate 2 (5-HT2A.mGlu2) receptor heteromerization and its psychoactive behavioral function. *J Biol Chem* 2012; **287**: 44301–44319.

48 Dunn HA, Zucca S, Dao M, Orlandi C, Martemyanov KA. ELFN2 is a postsynaptic cell adhesion molecule with essential roles in controlling group III mGluRs in the brain and neuropsychiatric behavior. *Mol Psychiatry* 2019; **24**: 1902–1919.

49 Alvaro-Bartolomé M, Esteban S, García-Gutiérrez MS, Manzanares J, Valverde O, García-Sevilla JA. Regulation of Fas receptor/Fas-associated protein with death domain apoptotic complex and associated signalling systems by cannabinoid receptors in the mouse brain. *Br J Pharmacol* 2010; **160**: 643–656.

50 Dudok B, Barna L, Ledri M, Szabó SI, Szabadits E, Pintér B *et al.* Cell-specific STORM super-resolution imaging reveals nanoscale organization of cannabinoid signaling. *Nat Neurosci* 2015; **18**: 75–86.

51 Honer WG, Kaufmann CA, Kleinman JE, Casanova MF, Davies P. Monoclonal antibodies to study the brain in schizophrenia. *Brain Res* 1989; **500**: 379–383.

52 Honer WG, Hu L, Davies P. Human synaptic proteins with a heterogeneous distribution in cerebellum and visual cortex. *Brain Res* 1993; **609**: 9–20.

SUPPLEMENTARY TABLES

Supplementary Table S1. Prior studies quantifying group II mGluRs in postmortem brains of subjects with schizophrenia (SZ) and controls (Con)

Receptor	Assay	Detection tool	No. of subjects	Brain area	Extraction method	Sample source	SZ *vs* Con findings	Effect of AP drugs	Year [Ref]
$mGlu_3R$	ISH	$mGlu_3R$ RNA probes	6 SZ, 10 Con	PFC (BA9/10/11)	Frozen sections	CBBL	No changes reported	No effects reported	1998 [28]
$mGlu_2R$, $mGlu_3R$	ISH	$mGlu_2R$ and $mGlu_3R$ RNA probes	12 SZ, 8 Con	Thalamus	Frozen sections	MSMC and BVAMC	No changes reported	No effects reported	2000 [29]
$mGlu_{2/3}R$	WB, IHC	Nonselective $mGlu_{2/3}R$ Ab (Chemicon AB1553)	20 SZ, 20 Con	DLPFC (BA46)	Membranal fraction	MEODC	No changes reported	All cases were on AP treatment. No association between mGluR amounts and the CPZE prescribed.	2002 [30]
$mGlu_{2/3}R$	WB	Nonselective $mGlu_{2/3}R$ Ab (Upstate, undisclosed Ab)	16 SZ, 9 Con	PFC (BA9/11/32/ 46) and STR	Total homogenate	MSMC and BVAMC	Increased $mGlu_{2/3}R$ protein in PFC	No effects reported	2005 [31]
$mGlu_3R$	WB	Non-commercial $mGlu_3R$ Ab	20 SZ, 35 Con	PFC (BA10)	Membranal fraction	CBBL	Reduced $mGlu_3R$ dimers	No effects reported	2007 [32]
$mGlu_3R$	qPCR, μarray	TaqMan cDNA probes	15 SZ, 15 Con	DLPFC (BA9)	Total homogenate	SNC	Reduced $mGlu_3R$ mRNA	No effects reported	2007 [33]
$mGlu_{2/3}R$	RLB, qPCR	[^{3}H]LY341495	25 SZ, 25 Con	DLPFC (BA9)	Membranal fraction	IVML	Reduced $mGlu_{2/3}R$ binding and $mGluR_2$ mRNA	Toxicological assessments found 18 AP+ cases. $mGlu_2R$ downregulation was observed in AP- subjects only	2008 [34]
$mGlu_2R$, $mGlu_3R$	qPCR	Selective exon spanning primer pairs	13 SZ, 13 Con	Cerebellum (lobule VIIa)	Total homogenate	MBC	Reduced $mGlu_2R$, but not $mGlu_3R$ mRNA	All cases were on AP treatment. Increased $mGlu_2R$ mRNA in haloperidol-, but not clozapine-treated rats	2008 [35]
$mGlu_2R$, $mGlu_3R$	ISH	Selective cDNA probes	14 SZ, 23 Con	DLPFC (BA46)	Frozen sections	MEODC	Increased $mGlu_2R$, but not $mGlu_3R$ mRNA in white, but not grey matter	11 cases were on AP. Inverse correlation between $mGlu_2R$ and lifetime dose of AP in the grey matter.	2008 [36]
$mGlu_3R$	qPCR	Selective cDNA primers	67 SZ, 105 Con	DLPFC, and Hipp	Total homogenate	NIMH, SAC	Inconsistent increase of $mGlu_3R$ mRNA in DLPFC	All cases were on AP treatment. AP effects were discarded based on haloperidol or clozapine treatments in rats	2008 [37]

$mGlu_2R$, $mGlu_3R$	WB	Abcam $mGlu_2R$ (ab52176) and $mGlu_3R$ (undisclosed) Ab	15 SZ, 15 Con	DLPFC (BA9), APT (BA38) and MC (BA4)	Total homogenate	DCMEO	Reduced mGlu3R, but not $mGlu_2R$ protein in DLPFC	Toxicological assays found 10 AP+ cases. No differences reported. No effects in AP treated rats	2009 [38]
$mGlu_3R$	ISH	Selective cDNA primers	20 SZ, 20 Con	AH	Frozen sections	MBC	No changes reported	No effects reported	2009 [39]
$mGlu_{2/3}R$	AR	[^{3}H]LY354740	37 SZ, 37 Con	DLPFC (BA46)	Frozen sections	NSWBTRC	No changes reported	No effects reported	2011 [40]
$mGlu_{2/3}R$	AR	[^{3}H]LY341495	15 SZ, 15 Con	ACC (BA24)	Frozen sections	SNC	No changes reported	No effects reported	2014 [41]
$mGlu_2R$, $mGlu_3R$	qPCR	Selective cDNA primers	15 SZ, 15 Con	DLPFC (BA9/46)	Total homogenate	SNC	No changes reported	Toxicological assays found 14 AP+ cases. $mGlu_{2/3}R$ expression did not correlate with CPZE prescriptions.	2012 [42]
$mGlu_3R$	WB	Selective $mGlu_3R$ Ab (Abcam ab166608)	63 SZ, 46 Con	STG (BA22)	Membranal fraction	NIMH	No changes reported	Toxicological assays found 27 AP+ cases, which did not differ from AP- cases in terms of receptor density	2016 [26]
$mGlu_{2/3}R$	RLB	LY379268 + [^{35}S]GTPγS	27 SZ, 27 Con	DLPFC (BA9)	Membranal fraction	IVML	Reduced $mGlu_{2/3}R$-dependent stimulation of Gq, but not Gi protein	No effects reported	2016 [43]
$mGlu_{2/3}R$	RLB	[^{3}H]LY341495	15 SZ, 15 Con	ACC (BA24), DLPFC (BA46), VC (BA17)	Membranal fraction	VBBN	No changes reported	No effects reported	2016 [44]
$mGlu_2R$	WB	Selective $mGlu_2R$ Ab (Abcam ab150387)	15 SZ, 15 Con	ACC (BA24), DLPFC (BA46)	Total homogenate	VBBN	No changes reported	No effects reported	2019 [45]

Abbreviations: Ab, antibody; ACC, anterior cingulate cortical; AH, anterior hippocampus; AP, antipsychotics; APT, anterior pole of the temporal; AR, autoradiography; BA, Brodmann's area; BVAMC, Bronx Veterans Administration Medical Center; CBBL, Cambridge Brain Bank Laboratory; ChIP, chromatin immunoprecipitation; CPZE, chlorpromazine equivalent; Con, controls; DCMEO, Dallas County Medical Examiners' Office; DLPFC, dorsolateral prefrontal cortex; Hipp, hippocampus; IHC, immunohistochemistry; ISH, In situ hybridization; IMVL, Instituto Vasco de Medicina Legal; MBC, Maryland Brain Collection; MC, motor cortex; MEODC, Medical Examiners' Office of the District of Columbia; mGluR; metabotropic glutamate receptor; MSMC, Mount Sinai Medical Center; NIMH, National Institutes on Mental Health; NSWBTRC, New South Wales Brain Tissue Resource Centre; PFC, prefrontal cortex; RLB, radioligand binding assay; SAC, Stanley Array Collection; SZ, shizophrenia; SNC, Stanley Neuropathology Consortium; STG, superior temporal gyrus; STR, striatum; VBBN, Victorian Brain Bank Network; VC, visual cortex; WB, Western blotting.

Supplementary Table S2. Individual demographic characteristics and blood toxicological findings[a] in subjects with schizophrenia and sex-, age-, and PMI-matched controls from Cohorts 1 (C1) and 2 (C2)

Pair	Subject	Dx	Sex	Age (y.o.)	PMI (h)	Storage (years)	Brain pH	Cause of death	Mechanism of death	Toxicology	
										Blood	Brain
C1-P1	C1-S1	SZ	M	31	14	15.6	n/a	Suicide	Fall from height	BZD	n/a
	C1-S2	Con	M	32	4	7.0	6.70	Accidental	Traffic accident	(negative)	n/a
C1-P2	C1-S3	SZ	M	48	20	15.3	n/a	Suicide	Jump into railway	(negative)	n/a
	C1-S4	Con	M	47	17	17.0	n/a	Natural	Heart failure	BZD	n/a
C1-P3	C1-S5	SZ	M	45	3	13.9	7.19	Suicide	Fall from height	BZD	n/a
	C1-S6	Con	M	48	7	17.0	n/a	Accidental	Traffic accident	(negative)	n/a
C1-P4	C1-S7	SZ	F	37	58	12.6	6.20	Suicide	Drug intoxication	Her, BZD	n/a
	C1-S8	Con	F	36	38	23.0	n/a	Homicide	Blade wound	Coc, Her, BZD	n/a
C1-P5	C1-S9	SZ	M	46	22	11.9	6.41	Suicide	Fall from height	(negative)	av.
	C1-S10	Con	M	46	24	10.7	6.48	Natural	Heart failure	(negative)	av.
C1-P6	C1-S11	SZ	F	37	26	9.2	5.76	Suicide	Fall from height	BZD	n/a
	C1-S12	Con	F	38	22	8.9	6.44	Accidental	Traffic accident	(negative)	n/a
C1-P7	C1-S13	SZ	M	48	11	9.3	6.43	Suicide	Fall from height	(negative)	n/a
	C1-S14	Con	M	49	8	8.7	6.20	Natural	Heart failure	(negative)	n/a
C1-P8	C1-S15	SZ	M	35	5	9.4	6.20	Suicide	Hanging	(negative)	n/a
	C1-S16	Con	M	37	6	24.7	n/a	Accidental	Traffic accident	EtOH	n/a
C1-P9	C1-S17	SZ	F	59	9	9.9	6.13	Natural	Heart failure	(negative)	n/a
	C1-S18	Con	F	57	4	13.0	n/a	Accidental	Traffic accident	(negative)	n/a
C1-P10	C1-S19	SZ	M	34	15	10.3	6.40	Natural	Heart failure	(negative)	n/a
	C1-S20	Con	M	34	17	6.9	6.70	Accidental	Traffic accident	(negative)	av.
C1-P11	C1-S21	SZ	M	52	7	10.6	6.32	Suicide	Fall from height	BZD	n/a
	C1-S22	Con	M	51	13	9.0	6.31	Accidental	Traffic accident	EtOH	n/a
C1-P12	C1-S23	SZ	M	44	7	16.0	6.72	Natural	Heart failure	Clt, Lvz, BZD	n/a
	C1-S24	Con	M	44	23	9.3	6.45	Accidental	Traffic accident	(negative)	av.
C1-P13	C1-S25	SZ	F	30	28	14.6	n/a	Suicide	Drug intoxication	Hal, BZD	n/a
	C1-S26	Con	F	30	18	9.9	6.60	Accidental	Traffic accident	(negative)	n/a
C1-P14	C1-S27	SZ	M	35	11	10.9	6.01	Natural	Heart failure	Clz, Clm, BZD	n/a
	C1-S28	Con	M	36	18	16.7	n/a	Accidental	Work accident	Bzg, EtOH	n/a
C1-P15	C1-S29	SZ	F	60	23	9.6	6.29	Natural	Peritonitis	Ami, Clz, BZD	n/a
	C1-S30	Con	F	60	48	10.1	6.80	Natural	Intracerebral hemorrhage	Crz	n/a
C1-P16	C1-S31	SZ	M	56	12	10.0	6.40	Natural	Heart failure	Olz, Clt	n/a
	C1-S32	Con	M	54	16	9.3	6.50	Accidental	Work accident	EtOH	n/a
C1-P17	C1-S33	SZ	M	41	11	7.3	6.15	Suicide	Blade wound	Clt, Qtp, BZD	n/a
	C1-S34	Con	M	41	14	10.2	6.81	Natural	Heart failure	(negative)	n/a
C1-P18	C1-S35	SZ	M	42	14	11.0	6.20	Suicide	Drug intoxication	Ris, BZD	n/a
	C1-S36	Con	M	43	20	8.6	6.48	Natural	Heart failure	(negative)	n/a
C1-P19	C1-S37	SZ	M	36	8	12.7	n/a	Suicide	Drug intoxication	Olz	av.
	C1-S38	Con	M	36	23	9.4	6.42	Accidental	Squashing	(negative)	n/a
C1-P20	C1-S39	SZ	F	28	22	14.0	n/a	Suicide	Fall from height	Ris	n/a
	C1-S40	Con	F	32	19	15.2	n/a	Accidental	Traffic accident	EtOH	n/a
C1-P21	C1-S41	SZ	F	48	17	14.1	n/a	Suicide	Fall from height	Olz, Clt	av.
	C1-S42	Con	F	50	11	10.7	6.21	Natural	Heart failure	(negative)	n/a
C2-P1	C2-S1	SZ	M	49	12	2.0	5.90	Accidental	Asphyxiation	BZD	av.
	C2-S2	Con	M	46	6	1.6	6.33	Accidental	Traffic accident	EtOH	av.
C2-P2	C2-S3	SZ	M	60	12	2.8	6.57	Suicide	Fall from height	BZD	av.
	C2-S4	Con	M	62	9	4.7	6.25	Natural	Cardiopulmonary insufficiency	(negative)	av.
C2-P3	C2-S5	SZ	F	67	22	3.6	5.80	Natural	Endometrial cancer	(negative)	av.
	C2-S6	Con	F	66	16	1.2	n/a	Natural	Myocardial infarction	(negative)	av.
C2-P4	C2-S7	SZ	M	34	23	8.8	6.32	Suicide	Fall from height	(negative)	av.
	C2-S8	Con	M	34	17	7.9	6.70	Accidental	Traffic accident	(negative)	av.
C2-P5	C2-S9	SZ	M	32	52	9.1	6.30	Natural	Cardiopulmonary insufficiency	BZD	av. [b]
	C2-S10	Con	M	32	4	2.9	6.20	Accidental	CO_2 intoxication	EtOH, Coc	av. [b]
C2-P6	C2-S11	SZ	F	53	18	9.3	6.58	Natural	Digestive hemorrhage	(negative)	av.
	C2-S12	Con	F	53	22	1.0	n/a	Natural	Cardiopulmonary insufficiency	(negative)	av.

C2-P7	C2-S13	SZ	M	32	21	9.4	6.65	Suicide	Fall from height	(negative)	av.
	C2-S14	Con	M	32	16	2.0	6.00	Accidental	Gas inhalation	(negative)	av.
C2-P8	C2-S15	SZ	M	43	5	1.0	n/a	Accidental	Traffic accident	Olz, Ser, BZD	av.
	C2-S16	Con	M	44	29	1.2	n/a	Accidental	Drowning	(negative)	av.
C2-P9	C2-S17	SZ	M	58	6	1.0	n/a	Natural	Hemorrhage	Clt	av.
	C2-S18	Con	M	57	3	2.4	6.30	Accidental	Squashing	(negative)	av.
C2-P10	C2-S19	SZ	M	51	18	1.0	n/a	Natural	Cardiopulmonary insufficiency	Pal, BZD	av.
	C2-S20	Con	M	50	2	2.0	6.10	Natural	Cardiopulmonary insufficiency	(negative)	av.
C2-P11	C2-S21	SZ	M	51	28	1.3	n/a	Natural	Myocardial infarction	Clt, Olz, BZD	av.
	C2-S22	Con	M	50	24	0.8	n/a	Natural	Myocardial infarction	THC	av.
C2-P12	C2-S23	SZ	M	58	24	1.3	n/a	Natural	Myocardial infarction	Pal, Cit, Gbp	av.
	C2-S24	Con	M	58	20	1.0	n/a	Natural	Myocardial infarction	THC	av.
C2-P13	C2-S25	SZ	M	34	21	1.5	6.60	Natural	Hemorrhage	Hal, BZD	av.
	C2-S26	Con	M	33	17	2.0	6.10	Accidental	Gas inhalation	(negative)	av.
C2-P14	C2-S27	SZ	M	58	16	2.0	5.70	Natural	CVD (aortic dissection)	Ari	av.
	C2-S28	Con	M	56	15	0.7	n/a	Natural	Cardiopulmonary insufficiency	(negative)	av.
C2-P15	C2-S29	SZ	M	60	17	2.0	6.10	Natural	Cardiopulmonary insufficiency	Qtp, Prx, BZD	av.
	C2-S30	Con	M	60	14	0.6	n/a	Natural	Myocardial infarction	(negative)	av.
C2-P16	C2-S31	SZ	F	46	12	2.8	6.50	Natural	Myocardial infarction	Qtp, Trz, BZD	av.
	C2-S32	Con	F	48	21	1.8	6.58	Natural	Cardiopulmonary insufficiency	BZD	av.
C2-P17	C2-S33	SZ	M	43	6	2.8	6.33	Suicide	Fall from height	Pal, Gbp, BZD	av.
	C2-S34	Con	M	44	21	1.3	7.23	Accidental	Traffic accident	(negative)	av.
C2-P18	C2-S35	SZ	F	50	14	2.8	6.30	Suicide	Fall from height	Ris, Qtp, BZD	av.
	C2-S36	Con	F	49	18	1.0	n/a	Natural	Cardiopulmonary insufficiency	THC	av.
C2-P19	C2-S37	SZ	M	63	20	3.2	6.70	Natural	Cardiopulmonary insufficiency	Clt, BZD	av.
	C2-S38	Con	M	63	20	1.0	n/a	Natural	Pneumonia	(negative)	av.
C2-P20	C2-S39	SZ	M	48	13	4.3	5.45	Suicide	Fall from height	Hal, Sul, Clz, BZD	av.
	C2-S40	Con	M	47	20	1.0	n/a	Natural	Intracerebral hemorrhage	(negative)	av.
C2-P21	C2-S41	SZ	M	60	7	4.7	5.74	Natural	Cardiopulmonary insufficiency	Pal	av.
	C2-S42	Con	M	60	19	8.2	6.37	Natural	Cardiopulmonary insufficiency	(negative)	av.
C2-P22	C2-S43	SZ	M	52	24	4.8	n/a	Natural	Digestive hemorrhage	Clt, Zip, BZD	av.
	C2-S44	Con	M	52	23	1.3	7.70	Natural	Myocardial infarction	(negative)	av.
C2-P23	C2-S45	SZ	M	52	11	4.9	n/a	Suicide	Blade wound	Clz, Pal, BZD	av.
	C2-S46	Con	M	51	16	3.3	6.40	Accidental	Traffic accident	(negative)	av.
C2-P24	C2-S47	SZ	F	52	10	5.0	n/a	Accidental	Asphyxiation	Qtp, Olz, BZD	av.
	C2-S48	Con	F	51	10	8.0	6.30	Natural	Cardiopulmonary insufficiency	(negative)	av.
C2-P25	C2-S49	SZ	M	73	17	5.0	n/a	Natural	Cardiopulmonary insufficiency	Ris, BZD	av.
	C2-S50	Con	M	74	23	0.7	n/a	Natural	Myocardial infarction	(negative)	av.
C2-P26	C2-S51	SZ	M	38	10	8.2	6.70	Homicide	Fall from height	Clz	av.
	C2-S52	Con	M	37	11	8.1	6.40	Accidental	Fall from height	EtOH, THC, Aph	av.
C2-P27	C2-S53	SZ	M	41	17	8.2	6.36	Homicide	Blade wound	Clz, BZD	av.
	C2-S54	Con	M	41	15	1.3	n/a	Natural	Myocardial infarction	(negative)	av.

Abbreviations: Ami, amisulpiride; Aph, amphetamine; Ari, aripiprazole; av., available; BZD, benzodiazepine (not specified); Bzg, benzoylecgonine; Cit, citalopram; Clm, clomipramine; Clt, clotiapine; Clz, clozapine; Coc, cocaine; CoD, cause of death; Crz, carbamazepine; Con, control; Dx, diagnosis; EtOH, ethanol; F, female; Gbp, gabapentin; Hal, haloperidol; Her, heroin; Lvz, levomepromazine; M, male; n/a, not available; Olz, olanzapine; Pal, paliperidone; PMI, postmortem interval; Prx, paroxetine; Qtp, quetiapine; Ris, risperidone; SZ, schizophrenia; Ser, sertraline; Sul, sulpiride; THC, tetrahydrocannabinol; Trz, trazodone; y.o., years old; Zip, ziprasidone.

[a] Only psychoactive drugs are indicated

[b] Cases with undetectable levels of antipsychotics in blood samples who had detectable levels of paliperidone in the brain

Supplementary Table S3. List of antibodies used for characterization and quantification of target proteins by WB

Target protein	Vendor	Cat. No.	Immunogen	Host	Clone	Subclass	Working dilution	Validation reference
$mGlu_2R$	Advanced Targeting Systems	AB-N32	GST fusion protein including residues 87-134 of rat $mGlu_2R$	Mouse	mG2Na-s	IgG2a	1:2,000	[46]
$mGlu_2R$	Abcam	ab15672	Undisclosed 47-amino acid peptide within the rat $mGlu_2R$ N-terminal domain	Mouse	mG2Na-s	IgG2a	1:1,000	[47]
$mGlu_2R$	Santa Cruz	sc-271655	Synthetic peptide mapping residues 407-536 of human $mGlu_2R$	Mouse	Not specified	IgG1	1:500	[48]
$mGlu_2R$	Discovery Antibodies	crb2005007	Undisclosed synthetic peptide within the human $mGlu_2R$ sequence	Rabbit	Polyclonal	-	1:500	-
$mGlu_3R$	Abcam	ab166608	Undisclosed synthetic peptide within the human $mGlu_3R$ sequence	Rabbit	EPR9009(2)	IgG	1:5,000	[26]
CB_1R	Abcam	ab23703	Synthetic peptide mapping residues 461-472 of human CB_1R	Rabbit	Polyclonal	-	1:1,000	[49]
CB_1R	ImmunoGenes	-	Synthetic peptide mapping residues 443-473 of human CB_1R	Rabbit	Polyclonal	-	1:500	[50]
D_2R	Chemicon	AB5084P	Undisclosed 27-aa peptide within the third cytosolic loop of human D_2R	Rabbit	Polyclonal	-	1:500	[25]
SYP	Locally produced	-	Purified synaptosomes from human brain	Mouse	EP10	IgG1	1:50*	[51]
SNAP25	Locally produced	-	Purified synaptosomes from human brain	Mouse	SP12	IgG1	1:50*	[52]
PSD95	Sigma	MABN68	Undisclosed recombinant peptide within the human PSD95 sequence	Mouse	K28/43	IgG2a	1:2,000	-
β-actin	Millipore	ab6276	β-actin synthetic peptide (residues 2-16)	Mouse	AC-15	IgG1	1:100,000	-
β-actin	Abcam	A8227	Synthetic peptide mapping an undisclosed human β-actin sequence	Rabbit	Polyclonal	-	1:10,000	-

*Prepared from non-purified cell culture medium supernatants

Supplementary Table S4. List of primers and Taqman probes used to quantify the expression (by RT-qPCR) or the load of HPTMs in promoter-bound histones H3 and H4 at the mGlu$_2$R (*GRM2*) and mGlu$_3$R (*GRM3*) coding genes (by ChIP-PCR) in postmortem human brain tissues

Gene	Method	Species	Primer sequences (or *Taqman assay ID*)	
			Forward	Reverse
GRM2	RT-qPCR	Human	CTTATGCGACCCATGGTGAT	GGTTGGCCACCTGGATG
GRM3	RT-qPCR	Human	ACGGCTCCATTCAACCCAAA	ACACGTTGTATCGCCCCATT
GRM2	ChIP-PCR	Human	GGGATTCAGCACCACGAG	CTCCTCCGTTCCTCCAGAC
GRM3	ChIP-PCR	Human	GCGAGGTGGTAGCAGAAAAG	CTTTTTCGCATTCTCCCAAA
GAPDH	RT-qPCR	Human	(*Hs99999905_m1*)	
RPS13	RT-qPCR	Human	(*Hs01945436_u1*)	

Supplementary Table S5. List of polyclonal antibodies used in chromatin immunoprecipitation (ChIP) assays.

ID	Vendor	Cat. No.	Target			Effect on gene expression	Host	Dilution in ChIP
			histone	lysine	PTM			
H3K4Me3	Millipore	07-473	H3	K4	Trimethylation	**Permissive**	Rabbit	1:333
H3K27Me3	Millipore	07-449	H3	K27	Trimethylation	**Repressive**	Rabbit	1:200
H3panAc	Millipore	06-559	H3	Global	Pan-acetylation	**Permissive**	Rabbit	1:200
H3K9Ac	Millipore	07-352	H3	K9	Acetylation	**Permissive**	Rabbit	1:200
H3K27Ac	Millipore	07-360	H3	K27	Acetylation	**Permissive**	Rabbit	1:167
H4K5Ac	Millipore	07-327	H4	K5	Acetylation	**Permissive**	Rabbit	1:100
H4K16Ac	Millipore	07-329	H4	K16	Acetylation	**Permissive**	Rabbit	1:200

Supplementary Table S6. Main ANCOVA results analyzing differences in cortical immunodensities of the indicated receptors between schizophrenia (SZ) and control (Con) groups. Two series of analyses were performed with schizophrenia subjects grouped altogether (left) or split into antipsychotic negative (AP-) or antipsychotic positive (AP+) cases (right), according to blood toxicology. A graphical representation of these results is provided in Supplementary Figure S2.

$mGlu_2R$

Con *vs* SZ|All

	Source	DF	SS	F-ratio	P-val
ANCOVA results	Model	4	20.3	7.18	<.001
	Error	91	64.5		
	C. Total	95	84.8		
Effect tests	Dx group	1	15.6	22.0	***<.001***
	Sex	1	2.28	3.22	0.076
	Age	1	2.47	3.49	0.065
	PMI	1	1.04	1.47	0.229

Con *vs* SZ|AP- *vs* SZ|AP+

	Source	DF	SS	F-ratio	P-val
ANCOVA results	Model	5	22.1	6.33	<.001
	Error	90	62.8		
	C. Total	95	84.8		
Effect tests	Dx group	2	17.3	12.4	***<.001***
	Sex	1	2.29	3.29	0.073
	Age	1	1.98	2.84	0.096
	PMI	1	1.45	2.07	0.153

	Source	Difference (95% CI)	P-val
Tuckey HSD *post hoc* test	Con *vs* AP-	0.55 (0.001–1.11)	***0.049***
	Con *vs* AP+	0.95 (0.49–1.42)	***<.001***
	AP- vs AP+	0.39 (-0.20–1.00)	0.264

$mGlu_3R$

Con *vs* SZ|All

	Source	DF	SS	F-ratio	P-val
ANCOVA results	Model	4	9.14	2.19	0.077
	Error	91	95.0		
	C. Total	95	104.2		
Effect tests	Dx group	1	0.97	0.93	0.338
	Sex	1	0.01	0.01	0.940
	Age	1	4.44	4.25	***0.042***
	PMI	1	3.97	3.80	0.054

Con *vs* SZ|AP- *vs* SZ|AP+

	Source	DF	SS	F-ratio	P-val
ANCOVA results	Model	5	19.4	4.11	0.002
	Error	90	84.8		
	C. Total	95	104.2		
Effect tests	Dx group	2	11.2	5.95	***0.004***
	Sex	1	0.01	0.01	0.929
	Age	1	6.09	6.47	***0.013***
	PMI	1	2.23	2.37	0.127

	Source	Difference (95% CI)	P-val
Tuckey HSD *post hoc* test	Con *vs* AP-	0.80 (0.164–1.45)	***0.010***
	Con *vs* AP+	0.16 (-0.37–0.70)	0.750
	AP- vs AP+	0.97 (0.270–1.67)	***0.004***

CB_1R

Con *vs* SZ|All

	Source	DF	SS	F-ratio	P-val
ANCOVA results	Model	4	6.18	1.76	0.144
	Error	91	79.9		
	C. Total	95	86.1		
Effect tests	Dx group	1	4.55	5.18	***0.025***
	Sex	1	1.16	1.33	0.253
	Age	1	0.14	0.16	0.690
	PMI	1	0.81	0.93	0.339

Con *vs* SZ|AP- *vs* SZ|AP+

	Source	DF	SS	F-ratio	P-val
ANCOVA results	Model	5	6.18	1.39	0.235
	Error	90	79.9		
	C. Total	95	86.1		
Effect tests	Dx group	2	4.56	2.57	0.083
	Sex	1	1.16	1.31	0.255
	Age	1	0.13	0.15	0.701
	PMI	1	0.82	0.92	0.340

	Source	Difference (95% CI)	P-val
Tuckey HSD *post hoc* test	Con *vs* AP-	0.42 (-0.20–1.04)	0.251
	Con *vs* AP+	0.44 (-0.08–0.96)	0.114
	AP- vs AP+	0.02 (-0.65–0.70)	0.996

D_2R

Con *vs* SZ|All

	Source	DF	SS	F-ratio	P-val
ANCOVA results	Model	4	26.4	4.39	0.003
	Error	91	136.9		
	C. Total	95	163.4		
Effect tests	Dx group	1	0.41	0.27	0.603
	Sex	1	0.29	0.19	0.661
	Age	1	6.80	4.52	***0.036***
	PMI	1	14.7	9.75	***0.002***

Con *vs* SZ|AP- *vs* SZ|AP+

	Source	DF	SS	F-ratio	P-val
ANCOVA results	Model	5	29.4	3.94	0.003
	Error	90	134.0		
	C. Total	95	163.4		
Effect tests	Dx group	2	3.36	1.13	0.328
	Sex	1	0.30	0.20	0.656
	Age	1	5.70	3.83	0.054
	PMI	1	12.48	8.38	***0.005***

	Source	Difference (95% CI)	P-val
Tuckey HSD *post hoc* test	Con *vs* AP-	0.19 (-0.61–1.00)	0.834
	Con *vs* AP+	0.32 (-0.35–1.00)	0.489
	AP- vs AP+	0.52 (-0.36–1.40)	0.341

Variables significantly associated with receptor immunodensities are highlighted in red. *Abbreviations*: ANCOVA, analysis of covariance; AP-/+, antipsychotic negative/positive; Con, control group; C. Total, corrected total; CB_1R, cannabinoid CB_1 receptor; CI, confidence interval; D_2R, dopamine D_2 receptor; DF, degrees of freedom; Dx, diagnosis; $mGlu_{2/3}R$, metabotropic glutamate receptor 2/3; PMI, postmortem interval; SZ, schizophrenia; SS, sum of squares.

Supplementary Table S7. Summary of the results obtained in sex-, age-, and PMI-controlled regression models predicting GPCR immunodensities in the DLPFC of subjects with schizophrenia (n = 30), using the predicted occupancy estimates for D_2R (*Model 1*), $5HT_{2A}R$ (*Model 2*), or D_2R and $5HT_{2A}R$ (*Model 3*) as independent variables.

**Model outcome** *Independent variable(s)*	***Model 1***		***Model 2***		***Model 3***	
	AdjR^2 or Stdβ	*P*-val	AdjR^2 or Stdβ	*P*-val	AdjR^2 or Stdβ	*P*-val
**$mGlu_2R$ immunodensity**	-0.072	-	-0.098	-	-0.095	-
Predicted D_2R occupancy	-0.161	0.434	-	-	-0.332	0.309
Predicted $5HT_{2A}R$ occupancy	-	-	-0.031	0.876	0.215	0.492
**$mGlu_3R$ immunodensity**	-0.033	-	0.283	-	0.336	-
Predicted D_2R occupancy	0.306	0.1358	-	-	-0.430	0.097
Predicted $5HT_{2A}R$ occupancy	-	-	0.606	**<.001***	0.924	**<.001***
**CB_1R immunodensity**	-0.072	-	-0.017	-	0.027	-
Predicted D_2R occupancy	-0.004	0.983	-	-	-0.438	0.158
Predicted $5HT_{2A}R$ occupancy	-	-	0.220	0.257	0.545	0.072
**D_2R immunodensity**	0.049	-	0.133	-	0.117	-
Predicted D_2R occupancy	0.163	0.399	-	-	-0.214	0.463
Predicted $5HT_{2A}R$ occupancy	-	-	0.316	0.084	0.474	0.099

Abbreviations: AdjR^2, model adjusted R^2 value; Stdβ, standardized beta estimate
*Statistically significant

SUPPLEMENTARY FIGURES AND LEGENDS

Supplementary Figure S1

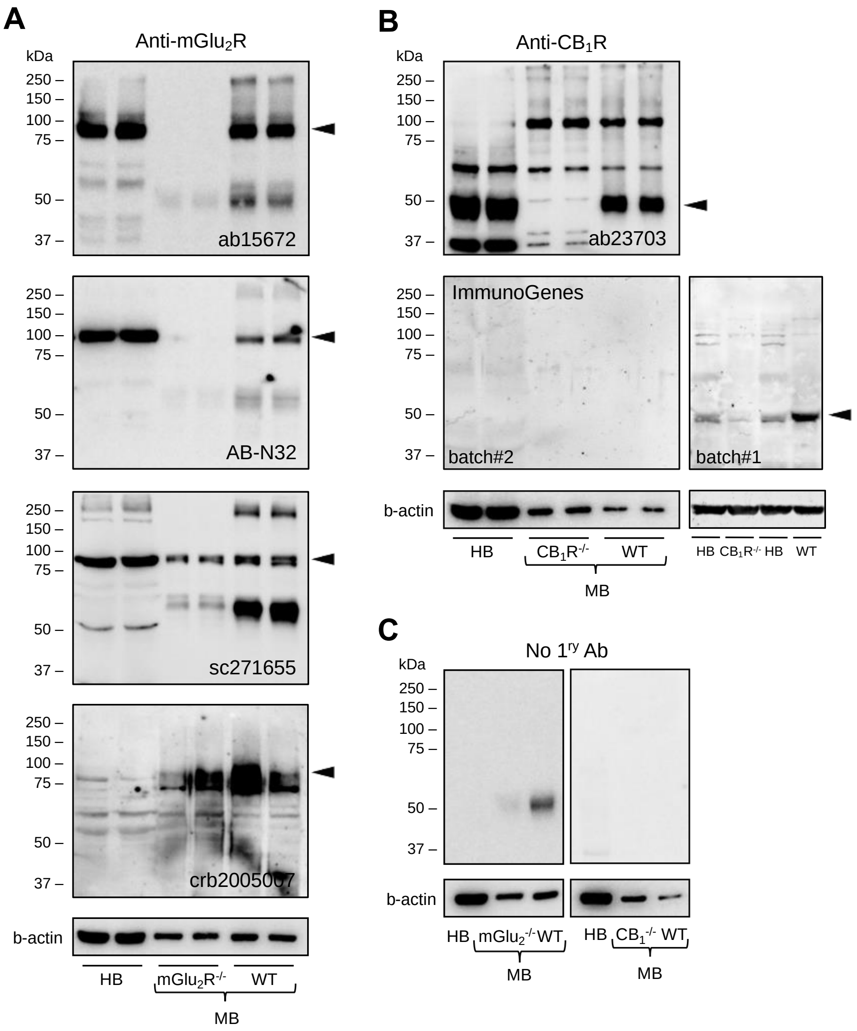

Characterization of all listed antibodies raised against (**A**) mGlu2R and (**B**) CB1R receptors (see Supplementary Table S3) in human and rodent brains, and validation in *Grm2* (mGlu2R$^{-/-}$) and *Cnr1* (CB1R$^{-/-}$) knockout mice. Images show representative immunoblots of human (HB), and wildtype (WT) or knockout mouse (MB) cortical samples loaded in duplicate onto 10% polyacrylamide gels, and resolved by standard SDS-PAGE, followed by immunoprobing with the commercial antibodies indicated at the bottom-right corner of the images. Arrowheads point at the expected molecular size of the protomeric forms of mGlu2 (~95 kDa) and CB1 (~50 kDa) receptors. (**C**) Experiments omitting the primary antibody showing minimal or no cross-reactivity of the HRP-conjugated secondary antibodies against mouse (left immunoblot) and rabbit (right immunoblot) IgG (heavy and light chains) with endogenous human and murine brain antigens. The weak signal in the anti-mouse immunoblot observed at ~55 kDa in mouse brain samples upon very long (i.e., 10 min) exposure times likely reflects the presence of circulating endogenous immunoglobulins. This signal was not present in human brain samples and therefore did not interfere with the reported measures in the study. All membranes were stripped and reprobed with anti-ß-actin antibody as a loading control. Molecular mass (in kDa) of SDS-PAGE prestained standards are shown on the left.

Supplementary Figure S2

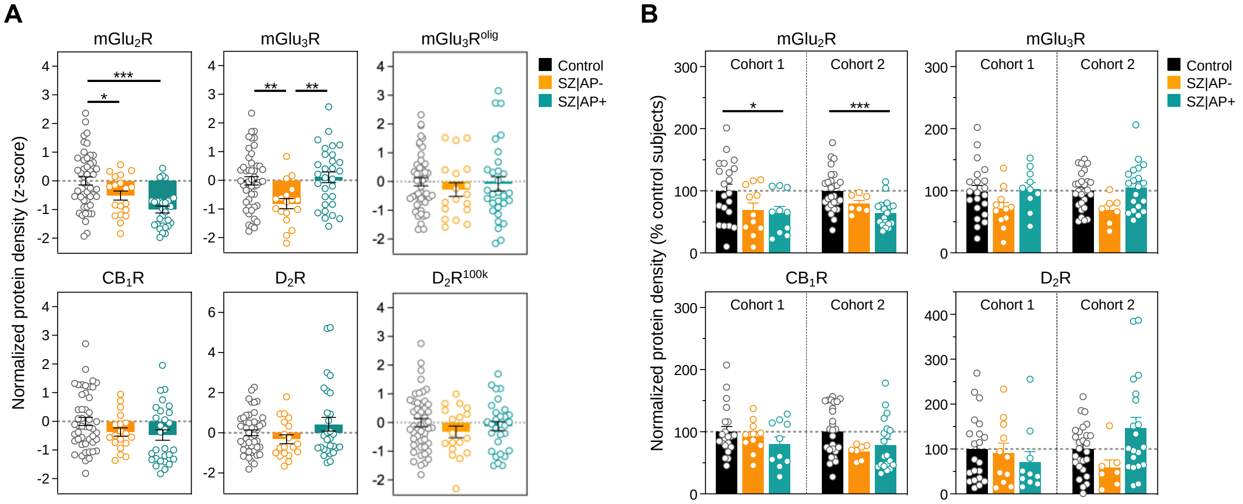

Bar plots representing ß-actin-normalized mGlu$_2$R, mGlu$_3$R (monomeric and oligomeric [mGlu$_3$R^{olig}] species), CB$_1$R, and D$_2$R (monomeric and 100 kDa [D$_2$R^{100k}] species) immunodensities in the DLPFC of controls subjects and schizophrenia cases with absence (SZ|AP-) or presence (SZ|AP+) of antipsychotic drugs in the blood at the time of death. Samples from Cohort 1 and Cohort 2 were represented altogether (**A**) or segregated (**B**). ANCOVA tests adjusting for age, sex and PMI, detected significant differences for mGlu$_2$R and mGlu$_3$R brain immunodensities (see Supplementary Table S6). *P < .05, **P < .01, ***P < .001, ANCOVA followed by Tuckey HSD *post hoc* test.
